# CD4 T cells mediate brain inflammation and neurodegeneration in a mouse model of Parkinson's disease

**DOI:** 10.1093/brain/awab103

**Published:** 2021-03-11

**Authors:** Gregory P Williams, Aubrey M Schonhoff, Asta Jurkuvenaite, Nicole J Gallups, David G Standaert, Ashley S Harms

**Affiliations:** Department of Neurology, Center for Neurodegeneration and Experimental Therapeutics, The University of Alabama at Birmingham, Birmingham, AL 35294, USA

**Keywords:** Parkinson disease, alpha-synuclein, T cells, neuroinflammation

## Abstract

α-Synuclein, a key pathological component of Parkinson's disease, has been implicated in the activation of the innate and adaptive immune system. This immune activation includes microgliosis, increased inflammatory cytokines, and the infiltration of T cells into the CNS. More recently, peripherally circulating CD4 and CD8 T cells derived from individuals with Parkinson’s disease have been shown to produce Th1/Th2 cytokines in response to α-synuclein, suggesting there may be a chronic memory T cell response present in Parkinson’s disease. To understand the potential effects of these α-syn associated T cell responses we used an α-synuclein overexpression mouse model, T cell-deficient mice, and a combination of immunohistochemistry and flow cytometry. In this study, we found that α-synuclein overexpression in the midbrain of mice leads to the upregulation of the major histocompatibility complex II (MHCII) protein on CNS myeloid cells as well as the infiltration of IFNγ producing CD4 and CD8 T cells into the CNS. Interestingly, genetic deletion of TCRβ or CD4, as well as the use of the immunosuppressive drug fingolimod, were able to reduce the CNS myeloid MHCII response to α-synuclein. Furthermore, we observed that CD4-deficient mice were protected from the dopaminergic cell loss observed due to α-syn overexpression. These results suggest that T cell responses associated with α-synuclein pathology may be damaging to key areas of the CNS in Parkinson’s disease and that targeting these T cell responses could be an avenue for disease modifying treatments.

## Teaser

Williams *et al*. report that α-synuclein overexpression in the mouse midbrain leads to infiltration of CD4 and CD8 T cells into the CNS. They show that CD4 T cells help mediate α-synuclein-induced myeloid inflammation and neurodegeneration, thereby providing insights into the neuroimmunology of Parkinson’s disease.

## Introduction

Neuroinflammation is an established component of Parkinson's disease, the most common neurodegenerative movement disorder affecting an estimated 1 million individuals in the USA alone.^1^ The neuroinflammatory hallmarks of Parkinson’s disease include reactive CNS myeloid cells,^2^^,^^3^ T cell infiltration into the CNS,^4^^,^^5^ and increased pro-inflammatory cytokines/chemokines in the blood, CSF, and brain parenchyma of patients with Parkinson’s disease.^6-8^ In terms of the T cell response associated with Parkinson’s disease, several studies have shown an overall decreased amount of circulating T cells in Parkinson’s disease patients,^9–11^ but an increase in pro-inflammatory T cell subsets over regulatory ones.^10^^,^^12^^,^^13^ Recent work has indicated that alpha-synuclein (α-syn), the key pathological protein dysfunctional in Parkinson’s disease, can serve as an activator of these circulating T cells.^14–16^ However, the link between α-syn pathology, T cells, and how these interactions contribute to the overall neuroinflammation and neurodegeneration observed in Parkinson’s disease remains unclear.

Antigen presentation via the major histocompatibility complex [MHC, human leukocyte antigen (HLA) in humans] and subsequent T cell interrogation is a process that has evolved to detect and eliminate pathogens. Through the interaction of factors including genetic predisposition and previous infections, this immune process can become dysfunctional and contribute to disease. For example, T cells have been shown to play a pivotal role in the establishment and severity of the debilitating CNS disease multiple sclerosis. During disease, T cells can misrecognize self-CNS antigens (i.e. myelin) and mount an immune response that uses the innate immune response against cells harbouring those antigens.^17^ It is possible that a similar process could be occurring in Parkinson’s disease in response to α-syn or some other CNS antigen as genetic analyses of Parkinson’s disease cohorts have identified polymorphisms and haplotypes of the *HLA* region that are associated with disease risk.^18–20^

Using an adeno-associated viral model to overexpress α-syn (AAV-SYN) in the substantia nigra pars compacta (SNpc) of mice, we have previously shown an important contribution of major histocompatibility complex II-positive (MHCII^+^) CNS myeloid cells to the α-syn-induced neuroinflammatory and neurodegenerative phenotypes.^21^^,^^22^ In those studies, we also observed an influx of T cells into the midbrains of α-syn-expressing mice, but their contribution to the model was not further investigated. To evaluate the role of T cells in the response to α-syn overexpression in the CNS, we used the AAV-SYN mouse model, several lines of genetically modified mice with defects in T cells, pharmacological inhibition of T cells, and a combination of immunohistochemistry/flow cytometry. We found that in the AAV2-SYN model, both CD4 and CD8 T cells infiltrate into the CNS parenchyma. Additionally, we observed that knockout or pharmacological inhibition of T cells, specifically CD4 T cells, was able to reduce MHCII expression on CNS myeloid cells as well as protect against TH^+^ neuron loss in the ipsilateral SNpc. The data presented here support a critical role for T cells in α-syn induced neurodegeneration, and suggests there may be potential in the use T cell immunotherapies as a treatment for Parkinson’s disease and other synucleinopathies.

## Materials and methods

### Mice

In this study, we used male and female C57BL/6 (#000664 Jackson Laboratories), *Tcrb*^−^^/^^−^ (B6.129P2-Tcrbtm1Mom/J, #002118 Jackson Laboratories), *Cd4*^−/−^ mice (B6.129S2-Cd4^tm1Mak^/J, #002663 Jackson laboratories), and *Cd8*^−/^^−^ mice (B6.129S2-Cd8a^tm1Mak^/J, #002665 Jackson Laboratories); mice were maintained on a congenic background. All research conducted on animals was approved by the Institutional Animal Care and Use Committee at the University of Alabama at Birmingham.

### AAV2-virus

Construction, purification, and validation of the original recombinant (r)AAV vectors, rAAV-CBA-IRES-EGFP WPRE (CIGW) and rAAV-CBA-SYNUCLEIN-IRES-EGFP-WPRE (CISGW) are described in previous publications.^23–25^ The CIGW and CISGW rAAV vectors used in this study were manufactured by the University of Iowa Viral Vector Core.

### Stereotaxic surgery

Stereotaxic surgery performed on male and female C57BL/6 (wild-type), *Tcrb*^−/^^−^, *Cd4*^−/^^−^, and *Cd8*^−/^^−^ mice was in accordance with previously described protocols.^21^^,^^22^^,^^26^ In brief, mice were deeply anaesthetized with a mixture of oxygen/isoflurane and unilaterally (immunohistochemistry and stereology) or bilaterally (flow cytometry) injected with 2 µl of AAV2-GFP or AAV2-SYN [2.6 × 10^12^ viral genomes/ml diluted in sterile phosphate-buffered saline (PBS)] into the SNpc at a rate of 0.5 µl/min, a rest time of 2 min, and a withdraw time of 2 min. Stereotaxic coordinates used for the SNpc were anterior-posterior −3.2 mm from bregma, mediolateral ±1.2 mm from midline, and dorsoventral −4.6 mm from dura. All surgical procedures and postoperative care protocols were followed and approved by the Institutional Animal Care and Use Committee at the University of Alabama at Birmingham.

### Immunohistochemistry

At 4 or 26 weeks post-viral transduction animals were deeply anaesthetized and transcardially perfused with a heparinized PBS solution, followed by 4% paraformaldehyde (PFA) and PBS solution. Brains were post-fixed for 6 h or overnight in 4% PFA/PBS and then cryoprotected in a 30% sucrose solution in PBS for 48 h. Brains were then frozen on dry ice and cryosectioned coronally on a sliding microtome with a cut thickness of 40 µm. Sections were collected serially throughout the SNpc, placed into a tissue collection solution (50% PBS/50% glycerol), and stored at −20°C until immunohistochemical staining.

For fluorescent staining, free-floating sections were washed in Tris-buffered saline (TBS), blocked in 5% of appropriate serum, and then labelled with anti-tyrosine hydroxylase (TH) (clone: EP1536Y, Abcam), anti-alpha-synuclein (phospho-serine129, pSer129, clone EP1536Y, Abcam), anti-GFAP (clone: D1F4Q, Cell Signaling), anti-Iba1 (polyclonal, Wako), anti-MHCI (clone: 28-14-8, eBioscience), anti-MHCII (clone: M5/114.15.2, eBiosciences), anti-CD68 (clone: FA-11, Bio-Rad), anti-CD3 (clone 17A2, eBioscience), anti-CD4 (clone RM4-5, BD Bioscience), anti-CD8 (clone 4SM15, eBioscience), or anti-IgG (polyclonal, Jackson Laboratories) antibodies in TBS plus Triton (TBS-T), lightly shaking overnight at 4°C. The next day, appropriate Alexa-conjugated secondary antibodies diluted in TBS-T (Life Technologies) were applied at room temperature for 2 h. Sections were then mounted onto plus-coated glass slides, and cover slips were applied with hard set mounting medium to preserve fluorescent signal (Vector Laboratories).

For diaminobenzidine (DAB) staining, free-floating sections were washed in TBS, quenched of endogenous peroxidases, blocked in 5% of appropriate serum, and then labelled with anti-tyrosine hydroxylase (clone: EP1536Y, Abcam) antibody in TBS-T, lightly shaking overnight at 4°C. The next day, the appropriate biotinylated secondary antibody (Vector Laboratories) was diluted in TBS-T and applied for 2 h at room temperature. R.T.U. Vectastain ABC reagent (Vector Laboratories) and DAB kit (SK-4100; Vector Laboratories) were used according to the manufacturer's instructions to develop horseradish peroxidase reactions. Sections were mounted onto Plus^TM^ coated glass slides, dehydrated, and coverslipped using Permount^TM^ mounting medium (Fisher).

### Confocal imaging

Confocal images were acquired using either a Leica TCS-SP5 laser scanning confocal microscope or a Nikon Ti2-C2 confocal microscope. Images were saved using Leica LASF software or Nikon NIS-Elements software. Images were then exported and processed using Adobe Photoshop and Adobe Illustrator.

### Mononuclear cell isolation and flow cytometry

Mononuclear cells were isolated 4 or 12 weeks post-transduction from the ventral midbrain of mice bilaterally transduced with AAV2-GFP or AAV2-SYN according to previously published methods.^22^^,^^27^ Briefly, midbrains were dissociated and digested with 1 mg/ml Collagenase IV (Sigma) and 20 µg/ml DNAse I (Sigma) diluted in RPMI 1640 (Sigma). Mononuclear cells were then separated out using a 30/70% Percoll^®^ gradient (GE) and passing the interphase layer through a 70 µm filter.

For intracellular cytokine staining, isolated mononuclear cells were stimulated with phorbol myristate acetate (PMA) (50 ng/ml, Fisher BioReagents) and ionomycin (750 ng/ml, Millipore Sigma) in the presence of GolgiStop^TM^ (1:1000, BD Biosciences) for 4 h at 37°C/5% CO_2_. For all staining, isolated cells were blocked with anti-Fcy receptor (clone 2.4G2 BD Biosciences) and then surfaced stained accordingly with fluorescent-conjugated antibodies against CD45 (clone 30-F11, eBioscience), CD11b (clone M1/70, BioLegend), MHCII (M5/114.15.2, BioLegend), Ly6C (clone HK 1.4, BioLegend), TCRβ (clone H57-597, BioLegend), CD4 (clone GK1.5, BioLegend), and CD8a (clone 53–6.7, BioLegend). A fixable viability dye was used to distinguish live cells from debris per manufacturer’s instructions (Fixable Near-IR LIVE/DEAD Stain Kit, Invitrogen). For intracellular transcription factor and cytokine staining, cells were further processed using the Foxp3/Transcription Factor Staining Kit (eBioscience) or the BD Cytofix/Cytoperm Staining Kit (BD Biosciences), respectively, and then stained accordingly with fluorescent-conjugated antibodies against FOXP3 (clone FJK-16s, eBioscience), T-bet (clone 4B10, BioLegend), GATA3 (clone 16E10A23, BioLegend), RORγt (clone Q31-378, BD Biosciences), IFNγ (clone XMG1.2, eBioscience), IL-4 (clone 11B11, BioLegend), IL-17a (clone eBio17B7, eBioscience), or IL-10 (clone JES5-16E3, BioLegend). Samples were analysed by flow cytometry using an Attune Nxt flow cytometer (Thermo Fisher Scientific) and FlowJo software (Tree Star).

### FTY720 treatment

Preliminary experiments were performed to calculate an average mouse cage daily water consumption to determine dosing of FTY720 (TCI America). Based on those results, the appropriate amount of FTY720 to achieve a dose of 1 mg/kg/day (based on mouse weight/water consumption) was dissolved in 100% ethyl alcohol (vehicle) and mixed into regular mouse drinking water. The water was replaced two to three times a week and mice were monitored for signs of dehydration, with male mice drinking 3.2 ± 0.1 ml/day and female mice drinking 2.3 ± 0.2 ml/day on average. Although FTY720-treated mice drank less water on average than their vehicle treated counterparts (Supplementary Fig. 5A), FTY720-treated mice water consumption rates were consistent with other reports.^28^^,^^29^ Mouse weight was monitored throughout the FTY720 treatment and despite significant reduction in blood lymphocytes, no differences between vehicle and fingolimod were observed (Supplementary Fig. 5A). General disposition of the mice was also routinely monitored throughout experiments and no abnormal behaviours were observed.

### Stereology

For TH neuron quantification in the SNpc using unbiased stereological analysis as previously published.^21^^,^^22^^,^^30^ In summary, TH-DAB stained SNpc tissue slides (see ‘Immunohistochemistry’ section) were coded and then analysed with an Olympus BX51 microscope and the MicroBrightfield software (MicroBrightField). Four to five sections encompassing the rostrocaudal extent of the SNpc, both ipsilateral and contralateral to the injection site, were quantified using the optical fractionator method within the StereoInvestigator software. TH-positive neurons within the contours of the SNpc were counted on a 100 μm × 100 μm grid with a 50 μm × 50 μm counting frame and an optical dissector height of 22 μm. Weighted section thickness was used to correct for variations in tissue thickness at varying sites. Brightfield images depicting TH neurons in the SNpc were taken on the Olympus BX51 microscope.

### Statistical analysis

Flow cytometry experiments used three to four independent samples per group, with two ventral midbrains pooled per sample (i.e. each experiment used a total of six to eight mice per group). Data were analysed using an unpaired *t*-test, or a two-way ANOVA with Tukey’s multiple comparison test. Graphs displayed the mean ± SEM. *P < 0.05, **P < 0.01 ***P < 0.0005, ****P < 0.0001.

### Data availability

The authors affirm that the findings of this manuscript are supported by the data therein. Additional information can be requested from the corresponding author.

## Results

### α-Synuclein expression in the SNpc leads to infiltration of CD4 and CD8 T cells

**Figure 1:**
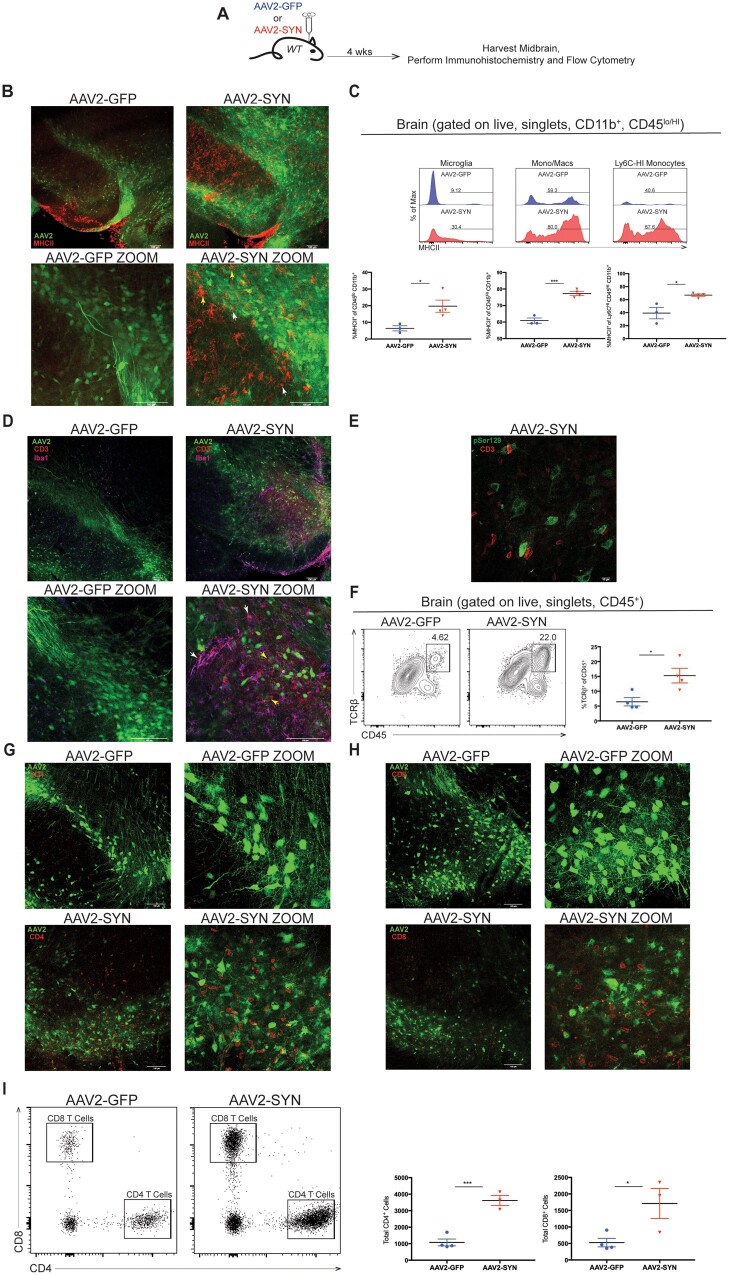
**α-Syn expression in the SNpc leads to infiltration of CD4 and CD8 T cells.** (**A**) C57BL/6J (wild-type, WT) mice 8–12 weeks of age received unilateral (immunohistochemistry) or bilateral (flow cytometry) stereotaxic injections of AAV2-GFP (control) or AAV2-SYN into the substantia nigra and were sacrificed for analysis 4 weeks later. (**B**) Immunohistochemistry 4 weeks post-transduction, eGFP (green) is visible in the ventral midbrain of AAV2-GFP and AAV2-SYN transduced animals. MHCII (red) is found to be upregulated in AAV2-SYN transduced animals over AAV2-GFP controls. Higher magnification (*bottom*) reveals the varying morphologies of MHCII^+^ CNS myeloid cells in AAV2-SYN injected mice. White arrowheads indicate circular monocyte-like morphology and yellow arrowheads indicate bushy microglia/macrophage morphology. (**C**) Mononuclear isolation and flow cytometry on isolated ventral midbrain tissues displaying the percentage of MHCII on resident microglia (CD45^lo^, CD11b^+^), monocytes/macrophages (Mono/Macs, CD45^HI^, CD11b^+^), and classical monocytes (Ly6C^HI^, CD45^HI^, CD11b^+^) in α-syn expressing mice compared to AAV2-GFP controls. Mean values are plotted ± SEM, unpaired *t*-test, *P < 0.05, ***P < 0.0005. (**D**) Representative confocal images depicting the presence of CD3^+^ (red) T cells in close relation to AAV2-SYN transduced neurons (green) and Iba1^+^ (magenta) CNS myeloid cells. Lower magnification panels, white arrowheads indicate close relation of T cells and CNS myeloid cells and yellow arrowheads indicate possible T cell/neuron interactions. (**E**) Representative image further depicting the close proximity of CD3^+^ T cells (red) to pSer129^+^ (green) AAV2-SYN transduced neurons in the substantia nigra of mice. (**F**) Flow cytometry plots depicting the gating used to quantify the T cell population in AAV2-SYN transduced mice compared to AAV2-GFP. Mean values are plotted ± SEM, unpaired *t*-test, *P < 0.05. (**G** and **H**) Representative confocal images show the accumulation of CD4 (**G**, red) and CD8 (**H**, red) T cells in AAV2-SYN transduced mice midbrains when compared to AAV2-GFP control. (**I**) Flow cytometry plots displaying the gating used to quantify the presence of CD4 and CD8 T cells in response to AAV2-SYN transduction. Mean values are plotted ± SEM, unpaired *t*-test, *P < 0.05, ***P < 0.0005. For immunohistochemistry experiments, *n* = 5–6 mice per group. For flow cytometry experiments, *n* = 3–4 (two mouse ventral midbrains pooled per *n*) per group. See Supplementary material for colourblind accessible versions of this figure.

We stereotaxically injected mice with either AAV2-GFP or AAV2-SYN and sacrificed them 4 weeks post-transduction for immunohistochemistry and flow cytometric analysis (Fig. 1A). When compared to AAV2-GFP control, AAV2-SYN expression resulted in the upregulation of MHCII (HLA class II in humans) protein on CNS myeloid cells in and around the SNpc (Fig. 1B). Higher magnification revealed the varying CNS myeloid morphologies of the AAV2-SYN associated CNS MHCII^+^ cells (Fig. 1B, enlarged image).

Flow cytometric analysis of AAV2-GFP or AAV2-SYN injected midbrain tissue allowed for stratification and quantification of the MHCII^+^ cells observed via immunofluorescent imaging (Fig. 1C). When gating on myeloid cells (CD45^+^, CD11b^+^; see Supplementary Fig. 1A for full gating strategies), increases in MHCII expression were observed on resident microglia (CD45^lo^, CD11b^+^), monocytes/macrophages (Mono/Macs, CD45^HI^, CD11b^+^), and classical monocytes (Ly6C^HI^, CD45^HI^, CD11b^+^) in the α-syn expressing mice compared to the controls. Furthermore, the number of total microglia was not significantly different between AAV2-GFP and AAV2-SYN animals, while there was a significant increase in the total number of macrophages and monocytes (Supplementary Fig. 2A). As it has been recently reported that astrocytes may also be able to express MHCII,^31^ we assayed whether this was true in response to AAV2-SYN. Although we observed clear GFAP expression in the midbrains of AAV2-SYN transduced mice, we did not observe any GFAP/MHCII double-positive cells (Supplementary Fig. 1B). These results indicate that the activated CNS myeloid response to α-syn is not solely from microglia or monocytes and their derived macrophages, but a combination of both.

We investigated whether the upregulation of MHCII by the CNS innate immune system was also associated with an accompanying T cell response. Immunofluorescent imaging of the ventral midbrain showed prominent labelling for CD3^+^ cells (a co-receptor critical for T cell activation) when compared to AAV2-GFP controls (Fig. 1D). Interestingly, these AAV2-SYN associated CD3^+^ T cells appear to be in close proximity with CNS Iba1^+^ myeloid cells as well as neurons (Fig. 1D, enlarged image). We have previously shown that α-syn overexpression in neurons leads to pathogenic pSer129 modifications of α-syn.^22^ Interestingly, CD3^+^ T cells could be observed alongside pSer129^+^ nigral neurons in AAV2-SYN transduced animals (Fig. 1E). We confirmed the T cell response with flow cytometric analysis and observed an expansion in the T cell compartment (CD45^HI^, TCRβ^+^) of AAV2-SYN animals over AAV2-GFP controls (Fig. 1F). This expanded CD3^+^/TCRβ^+^ T cell population responding to AAV2-SYN treatment was of both CD4 and CD8 origin as shown by immunohistochemistry (Fig. 1G and H) and flow cytometry (Fig. 1I). Given that the potential interaction between CD8 T cells and MHCI on neurons has been a focus of several studies,^32–34^ we performed more detailed visualization of CNS CD8 T cells and MHCI expression in AAV2-SYN transduced mice (Supplementary Fig. 3). We observed CD8 T cells in close proximity to both AAV2-SYN transduced neurons as well as Iba1^+^ CNS myeloid cells (Supplementary Fig. 3A). Furthermore, we found an increase in MHCI expression in AAV2-SYN transduced midbrains over GFP controls, and the MHCI expression could be found on the outside of TH-expressing cells (Supplementary Fig. 3B). We next explored which other cell types seemed to also be expressing MHCI in response to AAV2-SYN, and found that CNS myeloid cells (Iba1^+^), but not astrocytes (GFAP^+^), had double-positive cells for MHCI (Supplementary Fig. 3CandD). Broadly, these results indicate that the α-syn expression elicits an adaptive T cell response including infiltration of CD4 and CD8 T cells surrounding virally transduced neurons.

### α-Synuclein-responding T cells are predominantly IFNγ-producing CD4 T cells

The type of T cell response that occurs during an immune response is varied and is based on a variety of factors, such as the type of pathogen encountered and the tissue environment it is encountered in. Furthermore, the specific response of the T cell is critical in aiding in the clearance of the pathogen and, in cases of autoimmunity, the type of pathology T cells can cause.^35^

**Figure 2:**
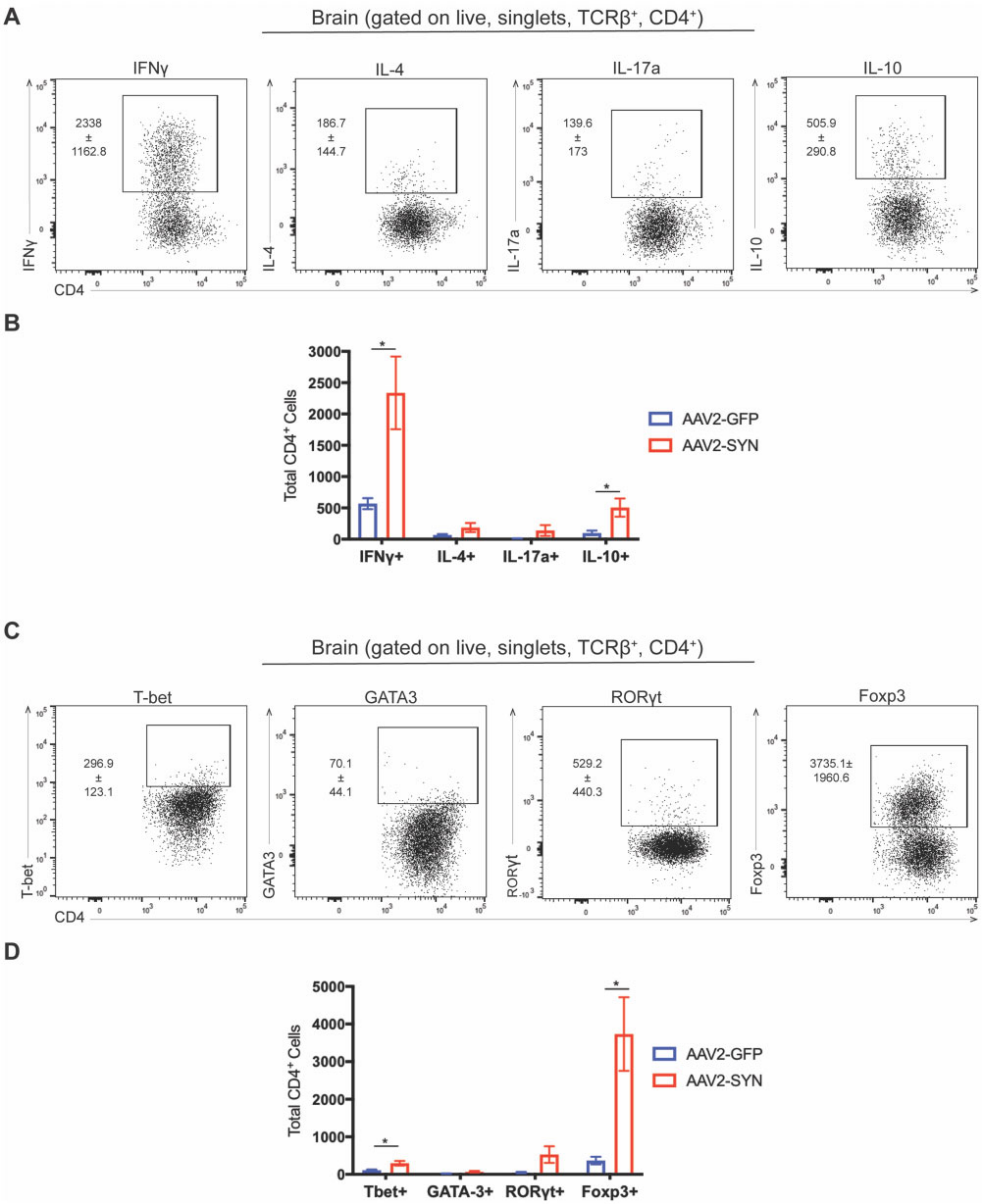
**α-Syn responding T cells are predominantly IFNγ producing CD4 T cells.** Intracellular cytokine and transcription factor staining was performed on C57BL/6J (wild-type, WT) mice 8–12 weeks of age that had received bilateral stereotaxic injections of AAV2-GFP (control) or AAV2-SYN into the substantia nigra and allowed to incubate for 4 weeks. (**A**) Representative flow plots depicting the gating used to quantify (**B**) Th1/2/17/Reg cytokines IFNγ, IL-4, IL-17a, and IL-10 in AAV2-GFP/SYN transduced mice. Mean values are plotted ± SEM, unpaired *t*-test, *P < 0.05. (**C**) Representative flow plots depicting the gating used to quantify (**D**) Th1/2/17/Reg master transcription factors T-bet, GATA3, RORγt, and Foxp3 in AAV2-GFP/SYN transduced mice. Mean values are plotted ± SEM, unpaired *t*-test, *P < 0.05. *n* = 4 (two mouse ventral midbrains pooled per *n*) per group.

We characterized the type of CD4 and CD8 T cell response observed in the AAV2-SYN mouse model. We performed both intracellular cytokine and transcription factor staining on mononuclear cells isolated from ventral midbrain tissue of mice 4 weeks post AAV2-GFP or AAV2-SYN transduction. Flow cytometric analysis of cytokine staining revealed an increase in the T helper 1 (Th1) and regulatory T cell (Treg) associated cytokines IFNγ and IL-10, but not in the Th2 and Th17 cytokines IL-4 and IL-17a (Fig. 2A and B). Of note, α-syn responding IFNγ^+^ CD4 T cells appeared to be the prevalent cytokine producing cell out of the cytokines assayed. We also observed an increase in the number of α-syn-responding IFNγ^+^ CD8 T cells (Supplementary Fig. 4A), but to a lesser degree than the CD4 population. The CD4/CD8 IFNγ and IL-10 responses observed were supported by transcription factor staining performed in tandem. We observed an increase in the Th1 and Treg associated transcription factors T-bet and Foxp3, but not in the Th2 and Th17 associated transcription factors GATA-3 and RORγt (Fig. 2C and D, Supplementary Fig. 4B). These data suggest that the increased T cell response seen in response to α-syn expression is predominantly made up of IFNγ producing CD4 T cells.

### Genetic or pharmacological depletion of T cells reduces CNS myeloid activation associated with α-synuclein expression

**Figure 3:**
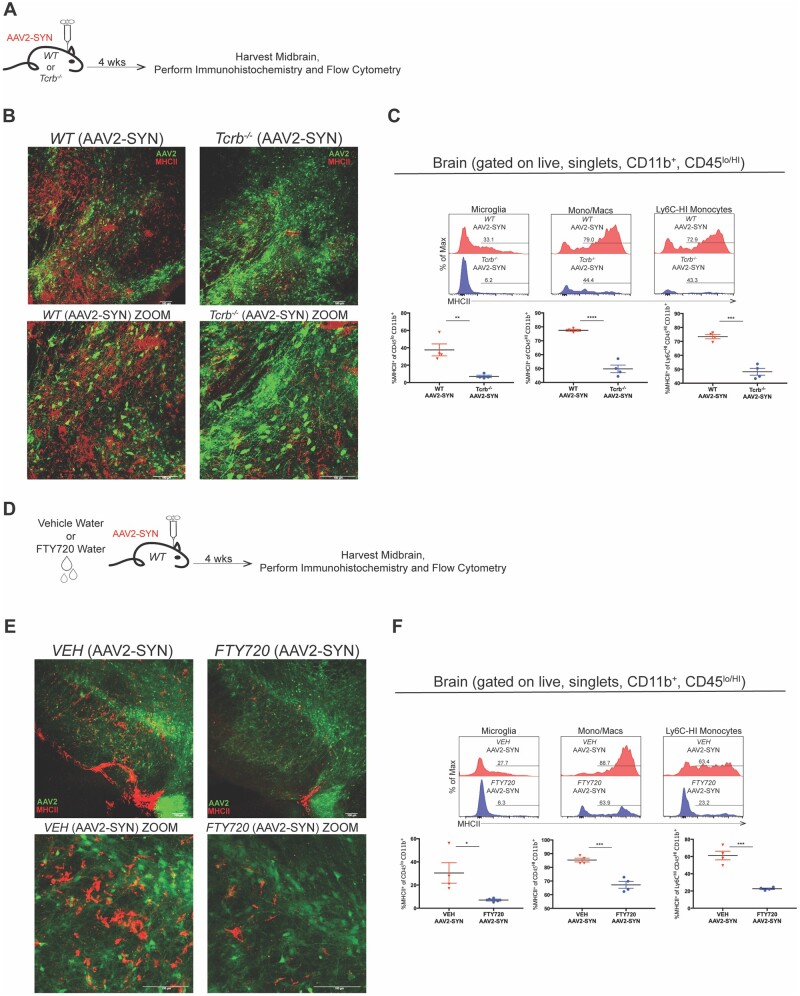
**Genetic or pharmacological depletion of T cells reduces CNS myeloid activation associated with α-syn expression.** (**A**) C57BL/6J (wild-type, WT) or *Tcrb*^−/^^−^ mice 8–12 weeks of age received unilateral (immunohistochemistry) or bilateral (flow cytometry) stereotaxic injections of AAV2-SYN into the substantia nigra and were sacrificed for analysis 4 weeks later. (**B**) Representative confocal images displaying MHCII (red) expression on CNS myeloid cells in AAV2-SYN transduced *Tcrb*^−/^^−^ mice compared to wild-type controls. (**C**) Flow cytometric gating used to quantify the MHCII expression on CNS microglia, mono/macs, and Ly6C-HI monocytes in *Tcrb*^−/^^−^ AAV2-SYN transduced mice compared to wild-type controls. Mean values are plotted ± SEM, unpaired *t*-test, **P < 0.01, ***P < 0.0005, ****P < 0.0001. (**D**) C57BL/6J (wild-type ) mice 8–12 weeks of age were pretreated with the immunosuppressive drug FTY720 or vehicle and then received unilateral (immunohistochemistry) or bilateral (flow cytometry) stereotaxic injections of AAV2-SYN into the substantia nigra and were sacrificed for analysis 4 weeks later. (**E**) Representative images displaying the reduction in MHCII (red) expression on CNS myeloid cells of FTY720/AAV2-SYN treated mice compared to VEH/AAV2-SYN controls. (**F**) Flow cytometric analysis displaying the MHCII expression on CNS microglia, mono/macs, and Ly6C-HI monocytes of FTY720/AAV2-SYN treated mice compared to vehicle treated controls. Mean values are plotted ± SEM, unpaired *t*-test, *P < 0.05, ***P < 0.0005. For immunohistochemistry experiments, *n* = 3–4 mice per group. For flow cytometry experiments, *n* = 4 (two mouse ventral midbrains pooled per *n*) per group. See Supplementary material for colourblind accessible versions of this figure.

Therapeutic strategies designed to disrupt T cell responses have been shown to be protective in preclinical disease models and have been the basis for treatments in human diseases such as multiple sclerosis or inflammatory bowel disease.^36^^,^^37^ With this in mind, we tested the hypothesis that the genetic knockout or pharmacological depletion of T cells could ameliorate the CNS myeloid activation associated with α-syn expression. We first transduced wild-type or *Tcrb*^−/^^−^ mice with AAV2-SYN and sacrificed them 4 weeks later for immunohistochemistry and flow cytometric analysis of the CNS immune response (Fig. 3A). As expected, we observed a significantly reduced amount of CD4 and CD8 T cells in the midbrains of AAV2-SYN transduced *Tcrb*^−/^^−^ mice compared to wild-type (Supplementary Fig. 5B). While wild-type AAV2-SYN-treated mice still had prominent MHCII staining in CNS myeloid cells with both monocyte and microglial/macrophage morphology in and around the SNpc, T cell-deficient *Tcrb*^−/^^−^ mice had noticeably less (Fig. 3B, enlarged images). Accordingly, we observed a reduction in MHCII expression on microglia, monocytes/macrophages, and Ly6C^HI^ monocytes in AAV2-SYN transduced *Tcrb*^−/^^−^ mice compared to wild-type (Fig. 3C). For total numbers of CNS myeloid populations, we found no differences in the amount of monocytes and macrophages infiltrating the CNS of *Tcrb*^−/^^−^ mice compared to wild-type (Supplementary Fig. 2B).

To pharmacologically deplete T cells, we chose the immunotherapeutic drug fingolimod (FTY720), a sphingosine receptor agonist that sequesters large amounts of lymphocytes in the lymph nodes thereby preventing their homing to peripheral tissues.^38^ We pretreated mice with FTY720 or vehicle in their drinking water for 5 days and analysed tail blood to confirm lymphopaenia (Supplementary Fig. 5C). We then transduced both groups with AAV2-SYN while concurrently administering vehicle or FTY720 treated water. Four weeks post-transduction, we analysed the CNS immune response (Fig. 3D). Although we had observed almost no CD4 or CD8 T cells in the blood of FTY720 treated animals, we still observed some CD4 and CD8 T cells in the ventral midbrains of those same animals 4 weeks post AAV2-SYN transduction, albeit to a lesser extent than vehicle-treated mice (Supplementary Fig. 5D). The presence of these FTY720 resistant CNS T cells raises the question of whether they might have originated from the CNS and not the circulation.^39^ When we looked at the CNS myeloid response following FTY720 treatment, we observed results similar to the genetic knockout of T cells: FTY720 treated mice had significantly less MHCII staining on CNS myeloid cells in their midbrains compared to the vehicle controls in response to α-syn (Fig. 3E). Likewise, we also observed a significant reduction in MHCII expression on microglia, monocytes/macrophages, and Ly6C^HI^ monocytes compared to vehicle in FTY720 treated mice when compared to vehicle control (Fig. 3F). In regard to the total numbers of CNS myeloid cells between vehicle and FTY720 mice, we found a significant reduction in the amount of monocytes/macrophages infiltrating the CNS of FTY720 mice compared to control, but no difference in microglia (Supplementary Fig. 2C). These data implicate that CNS infiltrating T cells from the periphery, responding to α-syn expression, have a significant impact in mediating the inflammatory CNS myeloid response.

### Genetic knockout of CD4, not CD8 T cells, ameliorates α-synuclein-mediated CNS myeloid activation

**Figure 4:**
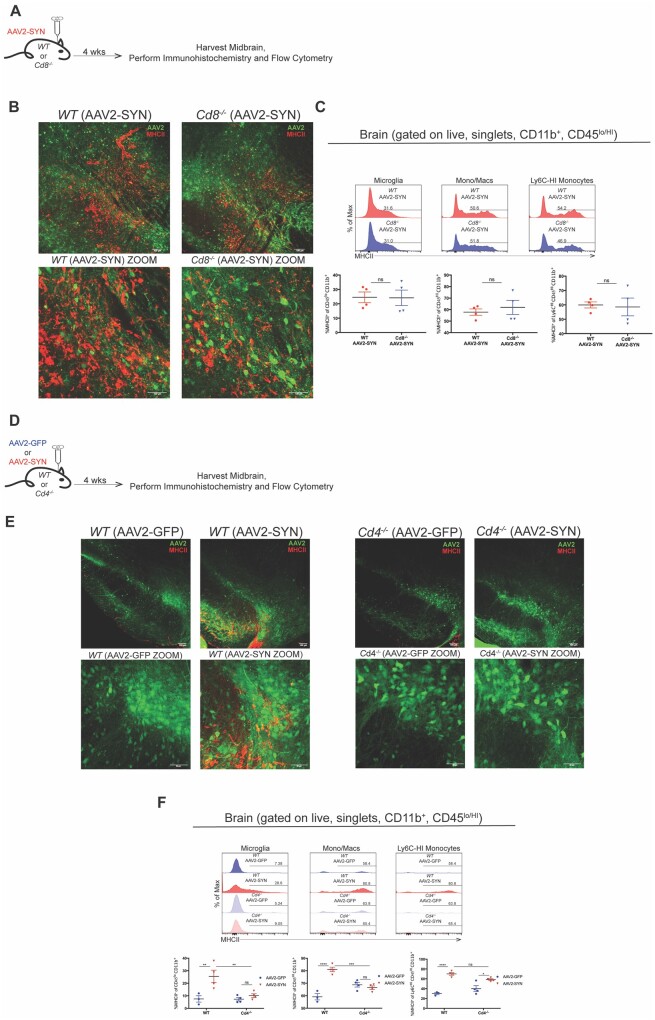
**Genetic knockout of CD4, but not CD8 T cells, ameliorates the CNS myeloid activation associated with α-syn expression.** (**A**) C57BL/6J (wild-type, WT) or *Cd8*^−/^^−^ mice 8–12 weeks of age received unilateral (immunohistochemistry) or bilateral (flow cytometry) stereotaxic injections of AAV2-SYN into the substantia nigra and were sacrificed for analysis 4 weeks later. (**B**) Representative confocal images depicting the MHCII (red) expression on CNS myeloid cells in AAV2-SYN transduced *Cd8*^−/^^−^ mice compared with wild-type controls. (**C**) Flow cytometry plots and associated quantification displaying the MHCII expression on CNS microglia, mono/macs, or Ly6C-HI monocytes between AAV2-SYN transduced wild-type or *Cd8*^−/^^−^ mice. Mean values are plotted ± SEM, unpaired *t*-test, ns = not significant. (**D**) C57BL/6J (wild-type) or *Cd4*^−/^^−^ mice 8–12 weeks of age received unilateral (immunohistochemistry) or bilateral (flow cytometry) stereotaxic injections of AAV2-GFP or AAV2-SYN into the substantia nigra and were sacrificed for analysis 4 weeks later. (**E**) Representative confocal images displaying MHCII (red) staining on CNS myeloid cells of AAV2-GFP/SYN (green) transduced wild-type/*Cd4*^−/^^−^ mice. (**F**) Flow cytometric gating and quantification displaying MHCII expression on CNS myeloid cells (microglia, mono/macs, and Ly6C-HI monocytes) of AAV2-SYN transduced *Cd4*^−/−^ mice compared to wild-type. Mean values are plotted ± SEM, two-way ANOVA with Tukey’s multiple comparison test, **P < 0.01, ***P < 0.0005, ****P < 0.000. For immunohistochemistry experiments, *n* = 5–6 mice per group. For flow cytometry experiments, *n* = 3–4 (two mouse ventral midbrains pooled per *n*) per group.

Given that genetic knockout or pharmacological depletion of CD4 and CD8 T cells is therapeutic in reducing the CNS myeloid response to AAV2-SYN, we next wanted to test which population was driving this inflammatory response. To examine this, we transduced wild-type or *Cd8*^−/^^−^ mice with AAV2-SYN and sacrificed after 4 weeks later for immunohistochemistry and flow cytometric analysis (Fig. 4A). In the SNpc, we observed that both wild-type and *Cd8*^−/^^−^ mice displayed similar MHCII expression on cells with microglial, macrophage, and monocyte morphologies in the vicinity of α-syn expression (Fig. 4B). Flow cytometric analysis revealed no difference in MHCII expression on the CNS myeloid populations between wild-type and *Cd8*^−/^^−^ mice following AAV2-SYN transduction (Fig. 4C). Additionally, we observed no difference in the amount of CNS CD4 T cells between AAV2-SYN treated wild-type and *Cd8*^−/^^−^ mice (Supplementary Fig. 6A). Given that AAV2-SYN treated mice lacking CD8 T cells still had an inflammatory myeloid response on par with wild-type mice, it appears that they are not the direct driver of the α-syn-driven myeloid response.

To test if CD4 T cells were the key mediator of this α-syn-driven innate response in the brain, we transduced wild-type and *Cd4*^−/^^−^ mice with either AAV2-GFP or AAV2-SYN and analysed the CNS myeloid response in the midbrain 4 weeks later. Similar to previous experiments, AAV2-SYN transduced wild-type mice had visible CNS myeloid activation in their midbrains compared to AAV2-GFP WT controls (Fig. 4E, top). Interestingly though, AAV2-SYN transduced *Cd4*^−/^^−^ mice had noticeably less myeloid activation, similar to *Tcrb*^−/^^−^ or FTY720 treated mice (Fig. 4E, bottom). Flow cytometric analysis of AAV transduced wild-type or *Cd4*^−/^^−^ mice showed that AAV2-SYN-injected wild-type mice displayed increased MHCII expression on microglia, monocytes/macrophages, compared to wild-type and *Cd4*^−/^^−^ GFP controls, and perhaps most importantly *Cd4*^−/^^−^-SYN mice (Fig. 4F). However, there was no difference in the Ly6C^HI^ MHCII levels between *Cd4*^−/^^−^ and wild-type mice transduced with AAV2-SYN. Of note, infiltrating CD8 T cells could still be observed in the CNS in wild-type AAV2-SYN transduced mice as well as *Cd4*^−/^^−^ mice (Supplementary Fig. 6B). We have previously reported on the increased presence of CD68-positive CNS myeloid cells in the ipsilateral midbrain of AAV2-SYN transduced mice.^23^^,^^25^^,^^40^ To determine if *Cd4*^−/−^mice expressed similar levels of CD68 after AAV2-SYN transduction we performed immunohistochemical staining and observed similar expression of CD68 between wild-type and *Cd4*^−/^^−^ animals, indicating that *Cd4*^−/^^−^ CNS myeloid cells may still have the same phagocytic capacity as their wild-type counterparts (Supplementary Fig. 6C). In addition to the reduced CNS MHCII response, when compared to wild-type, *Cd4*^−/^^−^ mice treated with AAV2-SYN appear to have markedly less midbrain IgG deposition, a humoral immune component previously associated with this model^21^^,^^25^ that has also been reported in human Parkinson’s disease^41^ (Supplementary Fig. 6D). In terms of the MHCI response, AAV2-SYN transduced *Cd4*^−/−^ mice had a similar increased expression as wild-type mice, while *Cd8*^−/−^ animals displayed reduced levels (Supplementary Fig. 6E). Taken together, these results indicate that CD4 T cells, and not CD8 T cells, are crucial in mediating the overall pro-inflammatory myeloid response to α-syn expression in the SNpc.

### Genetic knockout of CD4, but not CD8 T cells, ameliorates α-synuclein-induced neurodegeneration

**Figure 5:**
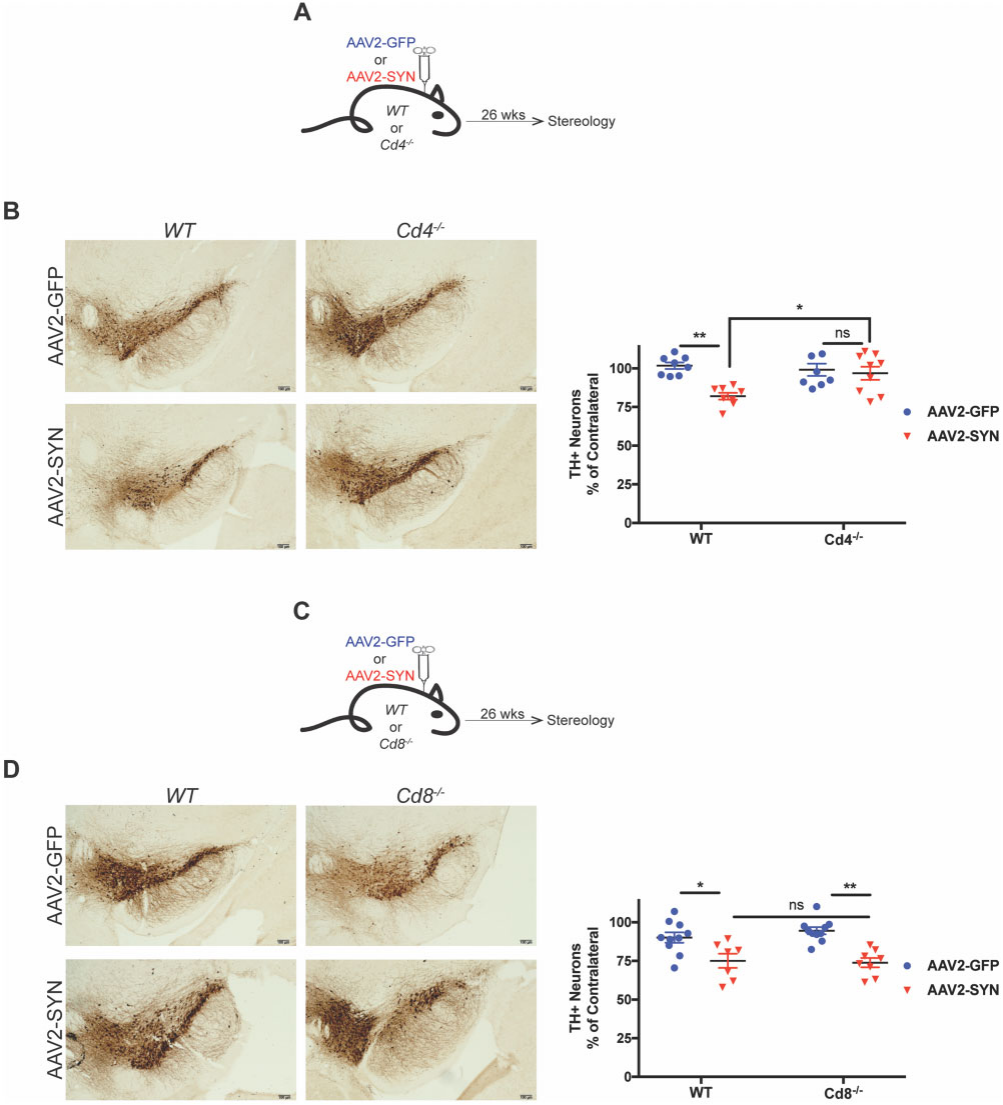
**Genetic knockout of CD4, but not CD8 T cells, ameliorates α-syn-induced neurodegeneration.** (**A**) C57BL/6J (wild-type, WT) or *Cd4*^−/^^−^ mice 8–12 weeks of age received unilateral stereotaxic injections of AAV2-GFP or AAV2-SYN into the substantia nigra and were sacrificed for unbiased stereology 26 weeks later. (**B**) Representative images depicting TH^+^ neurons (DAB, brown) in the ipsilateral substantia nigra of wild-type or *Cd4*^−/^^−^ 26 weeks post AAV2-GFP/SYN injection. Unbiased stereology was performed and counts are displayed on the *right*. Mean values are plotted ± SEM, two-way ANOVA with Tukey’s multiple comparison test, *P < 0.05, **P < 0.01. (**C**) C57BL/6J (wild-type, WT) or *Cd8*^−/^^−^ mice 8–12 weeks of age received unilateral stereotaxic injections of AAV2-GFP or AAV2-SYN into the substantia nigra and were sacrificed for unbiased stereology 26 weeks later. (**D**) Representative images depicting TH^+^ neurons (DAB, brown) in the ipsilateral substantia nigra of wild-type or *Cd8*^−/^^−^ 26 weeks post AAV2-GFP/SYN injection. Unbiased stereology was performed and counts are displayed on the *right*. Mean values are plotted ± SEM, two-way ANOVA with Tukey’s multiple comparison test, *P < 0.05, **P < 0.01. *n* = 8–10 mice per group.

We have previously shown that genetic knockout or silencing of MHCII attenuates TH^+^ neuron loss observed in response to AAV2-SYN transduction in the SNpc.^21^^,^^22^ After observing that CD4 T cells, and not CD8 T cells, are critical in promoting α-syn-induced MHCII expression on CNS myeloid cells, we wanted to determine whether this also applied to the nigral TH^+^ neuron loss. To do this, we transduced wild-type and *Cd4*^−/^^−^ mice with AAV2-GFP/AAV2-SYN and performed unbiased stereology in the SNpc 26 weeks later (Fig. 5A). Consistent with previous publications, we observed a significant loss of TH^+^ neurons in the ipsilateral SNpc compared to contralateral of wild-type mice transduced with AAV2-SYN over AAV-GFP animals (Fig. 5B). However, *Cd4*^−/^^−^-SYN mice did not display significant TH^+^ neuron loss when compared to their *Cd4*^−/^^−^-GFP counterparts. These data implicate that CD4 T cells not only drive the activated myeloid response to α-syn expression, but also the neurodegeneration associated with it.

As CD8+ T cells did not appear to be integral in the CNS myeloid response, we sought, to test if they were important for AAV2-SYN mediated neurodegeneration. Wild-type and *Cd8*^−/^^−^ mice were transduced with AAV2-GFP/AAV2-SYN and unbiased stereology was performed in the substantia nigra 26 weeks later (Fig. 5C). As observed in the previous experiment, wildt-type-SYN mice displayed a loss of SNpc TH^+^ neurons compared to wild-type-GFP animals. Similarly, *Cd8*^−/^^−^-SYN animals displayed a loss of nigral TH^+^ neurons when compared to their *Cd8*^−/^^−^-GFP counterparts, suggesting that CD8 T cells are not crucial in AAV2-SYN mediated neurodegeneration. Taken together, the data here support the hypothesis that CD4 T cells, and not CD8 T cells, are crucial in both the CNS myeloid response and the neurodegenerative processes that occur in the AAV2-SYN model of Parkinson’s disease.

## Discussion

In this study, we show that T cells, and more specifically CD4 T cells, contribute to the CNS innate immune cell response and dopaminergic neuron death in an α-syn driven mouse model of Parkinson’s disease. We observed both a CD4 and a CD8 T cell response as well as induction of MHCII^+^ on microglia, macrophages, and monocytes after the overexpression of α-syn in the SNpc. Interestingly, T cell-deficient mice (by genetic knockout or FTY720 treatment) displayed a dampened CNS myeloid responses to AAV2-SYN compared to wild-type controls. Lastly, we provide data that implicates IFNγ producing CD4 T cells as crucial to the CNS myeloid MHCII response and TH^+^ nigral neuron loss resulting from α-syn overexpression.

Previous work in the neurotoxin 6-hydroxydopamine (6-OHDA) and 1-methyl-4-phenyl-1,2,3,6-tetrahydropyridine (MPTP) models of Parkinson’s disease have provided important evidence of immune activation,^42^ and pointed to the role of CD4 T cells in mediating aspects of the inflammation and neurodegeneration in those models.^4^^,^^43–45^ Our studies extend upon this by showing a key role of T cells in not only neurotoxin-driven models, which are of uncertain relevance to human Parkinson’s disease, but also in an α-syn driven model which has much greater mechanistic validity with respect to the aetiology of human Parkinson’s disease. To that end, other work using the α-syn preformed fibril mouse model of Parkinson’s disease has implicated T cell infiltration as part of that model’s observed neuropathology.^46^^,^^47^ Furthermore, a study utilizing AAV-SYN overexpression in rats found that the use of an immunosuppressive drug (FK506) was able to reduce the amount of dopaminergic cell death in response to α-syn.^48^ Though it appears that some T cells may be promoting a damaging pro-inflammatory response in these models of Parkinson’s disease, it is important to mention that multiple studies have also detailed neuroprotective, Treg and natural killer (NK) responses in those same models.^46^^,^^49–51^ Overall, the findings in this study point to a potentially critical role of brain parenchymal CD4 T cells in Parkinson’s disease neurodegeneration.

It is important to note that although this study mainly implicates a CD4 T cell role in Parkinson’s disease, the CD8 T cell compartment has been shown to be dysregulated in human Parkinson’s disease,^4^^,^^16^^,^^32^^,^^52^ as well as to contribute to other preclinical models of the disease.^33^^,^^34^ While we did observe increased amounts of effector CD8 T cells in close association with α-syn infected neurons and CNS myeloid cells, knockout of CD8 T cells appeared to have no major effect on preventing the myeloid MHCII response or TH neuron loss. It could be that CD8 T cells by themselves do not significantly contribute to CNS neuroinflammation/neurodegeneration but do so when in conjunction with CD4 T cell responses, possibly through a MHCI mediated mechanism. To that point, MHCI expression in response to AAV2-SYN appeared to be partially mediated by CD8 T cells, but not CD4. Whatever the case, further characterization in other α-syn driven models and confirmation of an inflammatory T cell phenotype in human Parkinson’s disease tissue are needed to better support the link between CD8 T cells and Parkinson’s disease. Additionally, the potential role of B cells in Parkinson’s disease^41^^,^^53^ is an area of research that is deserving of attention and one only cursorily covered in this study with our observation of IgG deposition in response to AAV2-SYN and its amelioration in *Cd4*^−/^^−^ mice.

Using the AAV2-SYN model, we have demonstrated the importance of MHCII protein present on CNS antigen presenting cells in the neuroinflammatory/neurodegenerative responses to α-syn.^21^^,^^22^ The upregulation of this antigen machinery appears to be a reliable indicator of inflammation and possible neurodegeneration in the CNS, though further morphological characterizations of microglia at earlier time points to define their activation status better are needed. A key unresolved question is the nature of the antigens which create the link between α-syn, the immune system, and Parkinson’s disease. One possibility is that antigen presenting cells (microglia, CNS macrophages, and peripheral monocytes) may inappropriately load α-syn (or a modified form of this protein) onto their MHCII. Multiple factors including cellular machinery breakdown due to old age or genetic predisposition to overexpress α-syn could contribute to the access and misloading of pathogenic α-syn onto the MHCII. Furthermore, overexpression and certain haplotypes of *HLA*^16^^,^^18^^,^^19^ could also contribute to this theoretical misloading of α-syn onto the MHC. Circulating and surveilling T cells may bind to the presented α-syn (or some other neo-antigen) via their T-cell receptor (TCR) to produce an inflammatory adaptive immune response. This interaction would be possible if that T cell had escaped central tolerance or the antigen presented appeared novel due to modification—a common characteristic of pathogenic α-syn.^54^ This proposed TCR/α-syn/MHC binding is supported by CD4 and CD8 T cell-derived cytokine responses in Parkinson’s disease patient blood to α-syn and smaller peptides of the protein. Our work, however, does not confirm the direct TCR binding of CD4 T cells to CNS antigen presenting cells or the actual identity of the antigen being presented. These questions regarding Parkinson’s disease TCR and antigen identity should be the subject of future studies, both in humans and mice. To that point, a group has recently identified human Parkinson’s disease-specific TCRs which should be of great interest to the field.^55^

The pathological effects of excessive T cell cytokine release have been studied in the context of a variety of diseases and corresponding cell types including inflammatory bowel disease,^56^ allergic asthma,^57^ and multiple sclerosis.^17^ Some of these potential pathogenic cytokines include IL-4, IL-17a, TNF, and IFNγ. In this present study, we found excessive production of IFNγ, mainly from CD4 T cells, which was associated with an activated CNS myeloid compartment and TH neuron loss in the context of α-syn overexpression. This observation is consistent with work in other models that has shown IFNγ can activate microglia into an inflammatory phagocytic state,^58^ mediate the infiltration of blood circulating monocytes into the CNS,^59^ and cause damage in surrounding neurons.^60^ Conversely, we also observed an increase in IL-10 producing CD4 T cells in response to α-syn overexpression, which is suggestive of an accompanying Treg response alongside the predominant Th1 reaction in the brain. IL-10 signalling has been shown to play a critical role in restraining effector T cell responses in the context of resolving immune responses to pathogens and preventing the development of autoimmunity.^61^ In regard to human Parkinson’s disease, the Th1 cytokines IFNγ and TNF have been shown to be increased in Parkinson’s disease patient blood,^10^^,^^16^ while abnormal Treg responses from Parkinson’s disease patients are observed too.^12^^,^^13^ Taken together, these findings suggest that in Parkinson’s disease there may be an overactive Th1 T cell response that is worsened by a coinciding defective Treg response. However, some studies have shown contradictory results regarding the levels of IFNγ and other cytokines in the blood, CSF, or parenchyma in individuals with Parkinson’s disease compared to healthy controls.^8^ Furthermore, multiple groups have reported on the potential influence of Th17 cells in Parkinson’s disease,^5^^,^^10^ but this study did not find any significant differences in IL-17a/RORγt T cells. As is the case with most inflammatory diseases, it may be that in Parkinson’s disease there are multiple cytokines responses with different time courses that are involved in the inflammatory response. More work is needed to better describe these inflammatory signals, including when and where they occur during the disease progression of Parkinson’s disease; one recent study has shown that inflammatory T cell responses seem to be more prevalent in pre-symptomatic and early Parkinson’s disease stages.^14^

Presently, the treatment of Parkinson’s disease mainly revolves around the supplementation and regulation of dopamine or the direct electrical activation of motor circuits via deep brain stimulation. These treatments, while beneficial to restoring crucial motor functions impaired by Parkinson’s disease, do not halt or slow the actual progression of the disease. The need for transformative therapies for Parkinson’s disease will only to grow due to the projected incidence and associated economical burdens of Parkinson’s disease.^62^ Here we show, in a preclinical α-syn based model of Parkinson’s disease, that the administration of an immunotherapeutic T cell drug currently used to treat multiple sclerosis (fingolimod, Gilenya®) reduces the neuroinflammation associated with α-syn overexpression. For human Parkinson’s disease, it is important to note the potential significance of depleting the circulating immune system in a population with already reportedly lower lymphocyte counts.^10^ Critical next steps for the possible use of T cell therapies in Parkinson’s disease would be the further characterization of the signals those T cells produce and their effect in Parkinson’s disease. From there, multiple targeting paradigms already exist to dampen T cell responses in human disease which include their depletion, inhibiting their trafficking, or neutralizing the pro-inflammatory signals they are producing. Nonetheless, additional studies are required to confirm the effectiveness of these treatment rationales in other relevant models of Parkinson’s disease to determine if T cell therapy could be tested as a disease modifying treatment for human Parkinson’s disease.

## Supplementary Material

awab103_Supplementary_Data

## Abbreviations

α-syn: alpha-synuclein
MHCII: major histocompatibility complex II
SNpc: substantia nigra pars compacta
