# Supplementary material for: CD4 T cells mediate brain inflammation and neurodegeneration in a mouse model of Parkinson's disease
Source: Brain. 2021 Mar 11;144(7):2047–59. doi: 10.1093/brain/awab103 (PMC8370411; doi:10.1093/brain/awab103)
Supplement: awab103_Supplementary_Data [file awab103_supplementary_data.zip › brain-2020-01215-File007.pdf]

Supplementary Fig. 1:

A) Gating strategy for flow cytometric analysis. Single cell suspensions were stained for surface and/or intracellular markers, examined on an Attune Nxt flow cytometer, and analyzed using FlowJo software. Cells were gated on single, live cells before being gated into specific leukocyte populations as described in figures. Gray histogram overlays in MHCII gating figures represent fluorescent minus one (FMO) control samples that helped determine MHCII positive signal over background fluorescence. B) Representative immunohistochemistry in the midbrain of 4 weeks post-transduced AAV2-SYN (neurons, green) mice. Astrocytes (GFAP, red) are observed alongside MHCII^{+} CNS myeloid cells (blue). Higher magnification images (lower panel) reveal that there does not appear to be an overlap in GFAP/MHCII staining (merge).

Supplementary Fig. 2:

A) Quantification of flow cytometry on isolated ventral midbrain tissues displaying the numbers of resident microglia (CD45^{lo}, CD11b+), monocytes/macrophages (Mono/Macs, CD45^{HI}, CD11b^{+}), and classical monocytes (Ly6C^{HI}, CD45^{HI}, CD11b^{+}) in α-syn expressing mice compared to AAV2-GFP controls, B) compared to AAV2-SYN transduced *Tcrb*$^{-/-}$, and C) comparing vehicle or FTY720 treated AAV2-SYN mice. Mean values are plotted ± SEM, unpaired t-test, *p < 0.05, **p < 0.01. n=3-4 (2 mouse ventral midbrains pooled per n) per group.

Supplementary Fig. 3:

A) Representative immunohistochemistry in the midbrain of 4 weeks post-transduced AAV2-SYN WT mice. CD8 T cells (red) can be observed in close association with SYN transduced neurons (green) as well as Iba1^{+} CNS myeloid cells (blue). B) Immunohistochemistry of AAV2-GFP or AAV2-SYN injected WT mice 4 weeks post transduction. AAV2-SYN (left panel) mice display increased MHCI (red) expression in and around AAV2 (green) transduced neurons, including dopamine neurons (TH, blue) compared to GFP control mice. Higher magnification (right panels) images better detail the MHCI expression surrounding dopamine neurons in the substantia nigra. C) IHC of WT mice transduced with AAV2-SYN showing the MHCI (red) staining in relation to Iba1^{+}

cells (blue). Higher magnification (zoom) shows what appears to be overlap of MHCI (red) and Iba1 (blue) staining, whereas in D) WT mice stained with the astrocyte marker GFAP (blue), there does not seem to be overlap between GFAP and MHCI (red).

Supplementary Fig. 4:

Intracellular cytokine and transcription factor staining was performed on C57BL/6J (*WT*) mice 8-12 weeks of age that had received bilateral stereotaxic injections of AAV2-GFP (control) or AAV2-SYN into the substantia nigra and allowed to incubate for 4 weeks. A) Representative flow plots depicting the gating used to quantify IFNγ expression in AAV2-GFP/SYN transduced mice. B) Representative flow plots depicting the gating used to quantify T-bet expression in AAV2-GFP/SYN transduced mice. Mean values are plotted ± SEM, unpaired t-test, *$p < 0.05$. n=4 (2 mouse ventral midbrains pooled per n) per group.

Supplementary Fig. 5:

A) Weight change (left panels) and water consumption data (right panels) of WT mice receiving 1mg/kg/day of FTY720 or vehicle in their drinking water for the duration of experiments in Fig. 3D, n=8 mice per group. Mean values are plotted ± SEM, unpaired t-test, *$p < 0.05$. B) Flow cytometric gating used to quantify the amount of CD4 and CD8 T cells in the midbrains of 4 week AAV2-SYN transduced *Tcrb*$^{-/-}$ or *WT* mice. Mean values are plotted ± SEM, unpaired t-test, *$p < 0.05$, **$p < 0.01$. B) Flow cytometric gating used to quantify the amount of CD4 and CD8 T cells in the blood of vehicle or FTY720 treated *WT* mice (5 days post treatment). Mean values are plotted ± SEM, unpaired t-test, ***$p < 0.0005$, ****$p < 0.0001$. C) A) Flow cytometric gating used to quantify the amount of CD4 and CD8 T cells in the midbrains of 4 week AAV2-SYN transduced *WT* mice also treated with either vehicle or FTY720. Mean values are plotted ± SEM, unpaired t-test, *$p < 0.05$, ***$p < 0.0005$. n=3-4 (2 mouse ventral midbrains pooled per n) per group.

Supplementary Fig. 6:

A) Flow cytometric gating used to quantify the amount of CD4 T cells in the midbrains of 4 week AAV2-SYN transduced *Cd8*$^{-/-}$ or *WT* mice. Mean values are plotted ± SEM, unpaired t-test, ns=not significant. B) Flow cytometric gating used to quantify the amount of CD8 T cells in the midbrains of 4 week AAV2-GFP/SYN transduced *Cd4*$^{-/-}$ or *WT* mice. Mean values are plotted ± SEM, two-way ANOVA with Tukey's multiple comparison test, *p < 0.05, **p < 0.01. n=3-4 (2 mouse ventral midbrains pooled per n) per group. C) Representative immunohistochemistry in the midbrain of 4 weeks post-transduced AAV2-SYN WT and *Cd4*$^{-.-}$ mice to show the levels of the phagocytic marker CD68 (red) in Iba1^{+} (blue) CNS myeloid cells. D) Images displaying IHC of WT-GFP, WT-SYN, and *Cd4*$^{-/-}$-SYN transduced mice and the IgG deposition associated with them (red). Higher magnification (bottom panel) of just WT-SYN reveals that IgG staining surrounds some transduced (green), TH^{+} (blue) neurons. E) Representative IHC images of WT-SYN, *Cd4*$^{-/-}$-SYN, and *Cd8*$^{-/-}$-SYN animals 4 weeks post-transduction (green) and their associated MHCI expression (red).

A

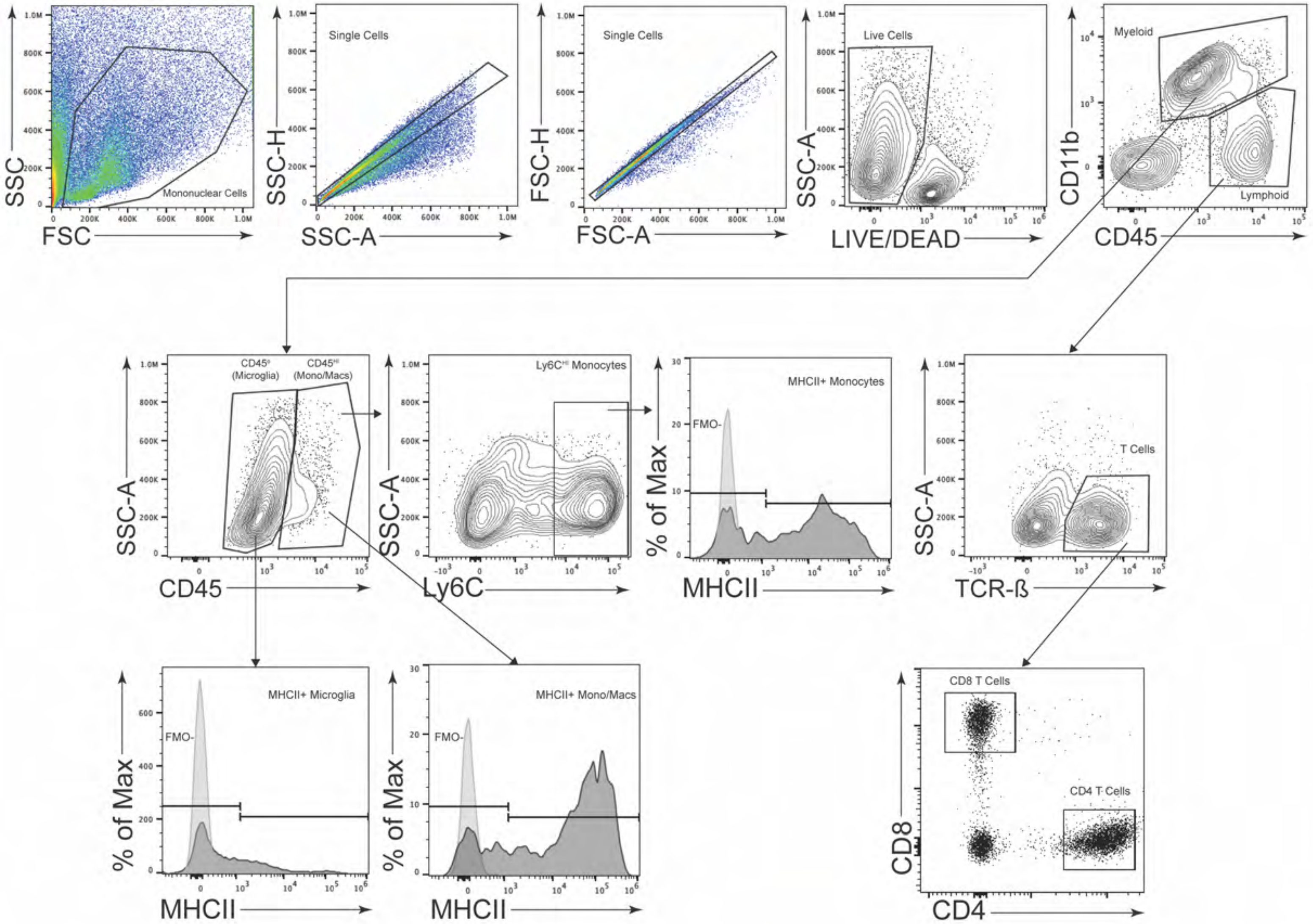
Mononuclear Cells
SSC
FSC
Single Cells
SSC-H
SSC-A
Single Cells
FSC-H
FSC-A
Live Cells
SSC-A
LIVE/DEAD
Myeloid
Lymphoid
CD11b
CD45
CD45^{lo} (Microglia)
CD45^{Hi} (Mono/Macs)
SSC-A
CD45
Ly6C^{Hi} Monocytes
SSC-A
Ly6C
MHCII+ Monocytes
FMO-
% of Max
MHCII
T Cells
SSC-A
TCR-ß
MHCII+ Microglia
FMO-
% of Max
MHCII
MHCII+ Mono/Macs
FMO-
% of Max
MHCII
CD8 T Cells
CD4 T Cells
CD8
CD4

B

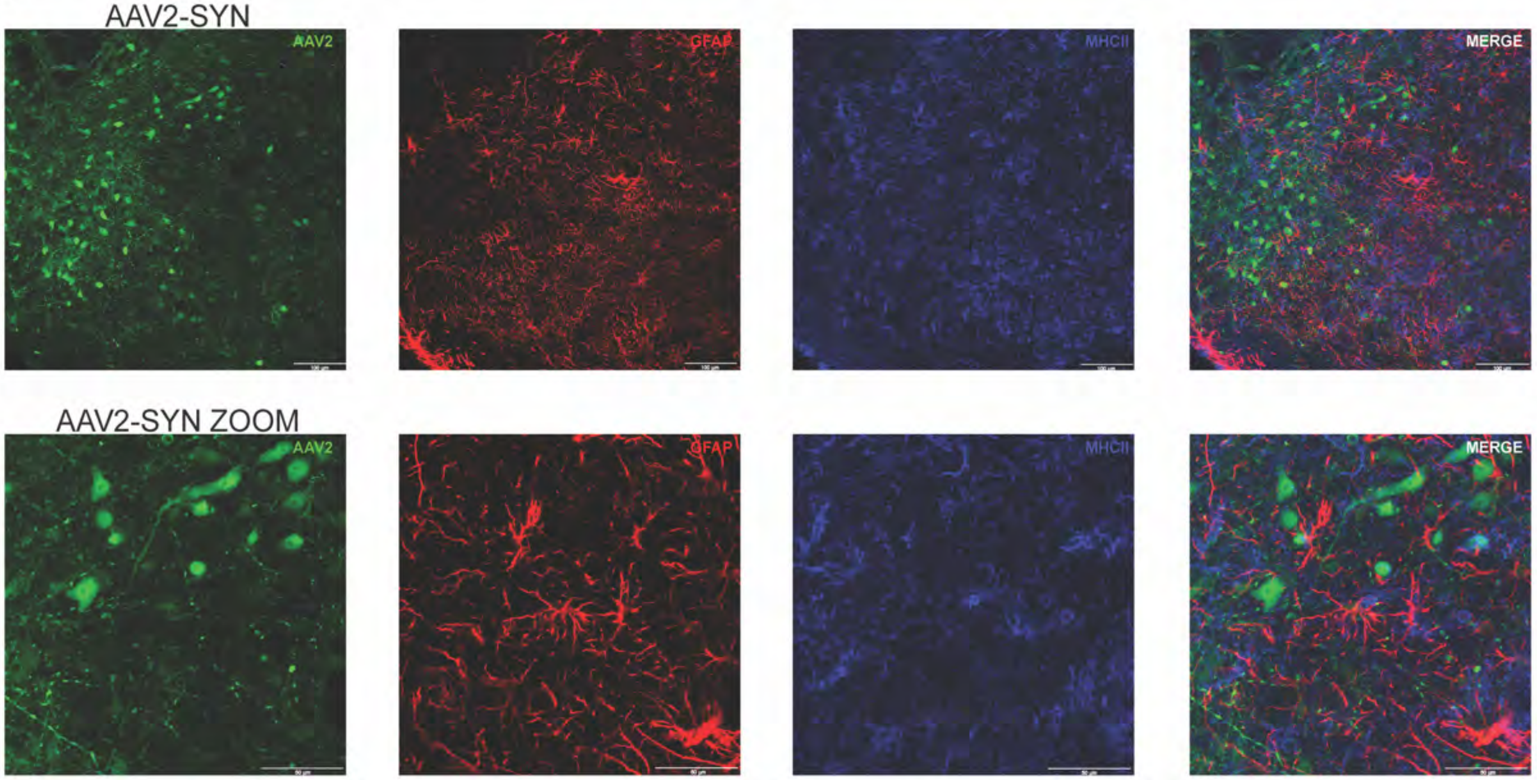
AAV2-SYN
AAV2
GFAP
MHCII
MERGE
AAV2-SYN ZOOM
AAV2
GFAP
MHCII
MERGE

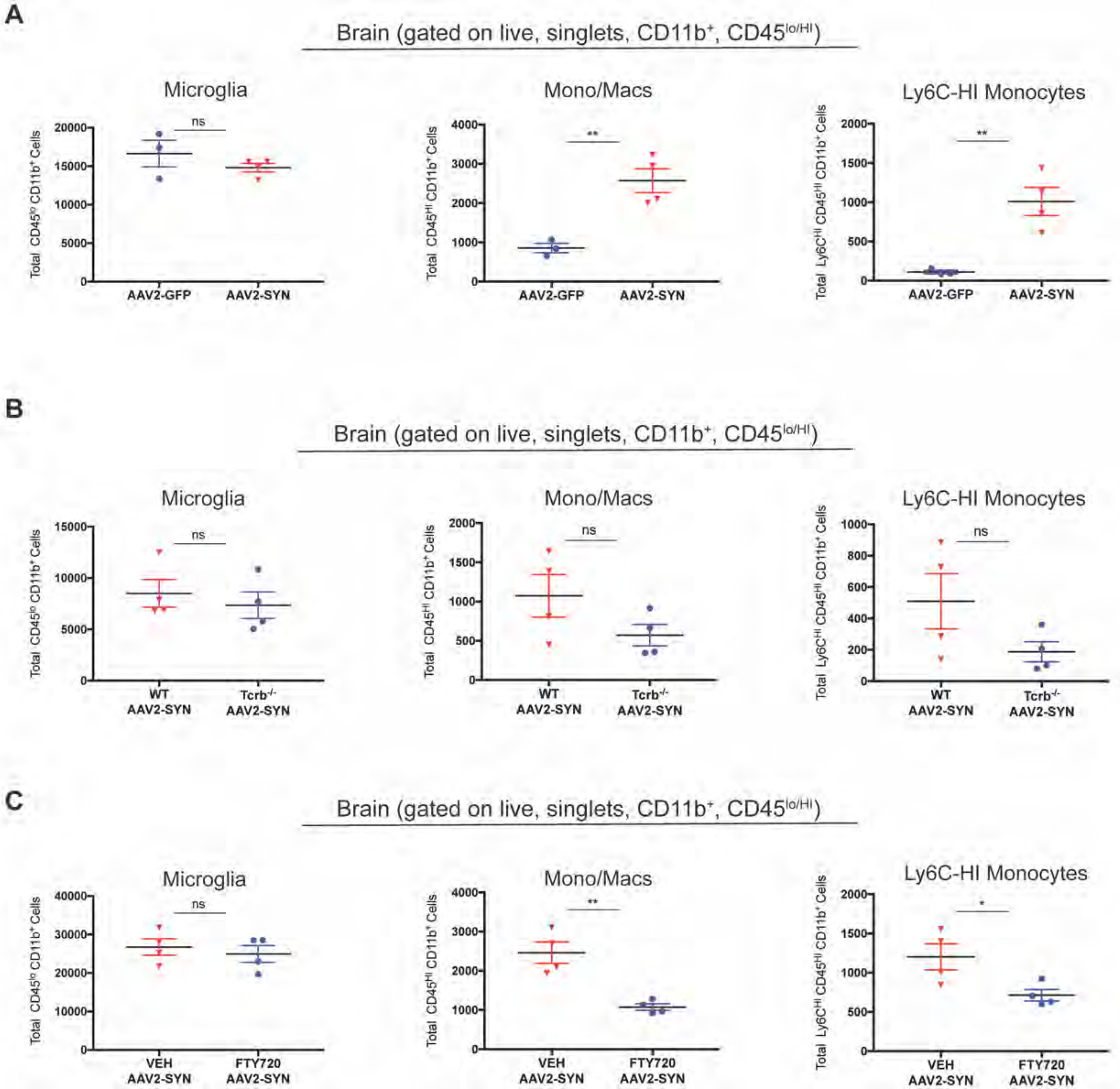
A
Brain (gated on live, singlets, CD11b+, CD45lo/HI)
Microglia
Total CD45lo CD11b+ Cells
ns
AAV2-GFP
AAV2-SYN
Mono/Macs
Total CD45HI CD11b+ Cells
**
AAV2-GFP
AAV2-SYN
Ly6C-HI Monocytes
Total Ly6CHI CD45HI CD11b+ Cells
**
AAV2-GFP
AAV2-SYN
B
Brain (gated on live, singlets, CD11b+, CD45lo/HI)
Microglia
Total CD45lo CD11b+ Cells
ns
WT AAV2-SYN
Tcrb-/- AAV2-SYN
Mono/Macs
Total CD45HI CD11b+ Cells
ns
WT AAV2-SYN
Tcrb-/- AAV2-SYN
Ly6C-HI Monocytes
Total Ly6CHI CD45HI CD11b+ Cells
ns
WT AAV2-SYN
Tcrb-/- AAV2-SYN
C
Brain (gated on live, singlets, CD11b+, CD45lo/HI)
Microglia
Total CD45lo CD11b+ Cells
ns
VEH AAV2-SYN
FTY720 AAV2-SYN
Mono/Macs
Total CD45HI CD11b+ Cells
**
VEH AAV2-SYN
FTY720 AAV2-SYN
Ly6C-HI Monocytes
Total Ly6CHI CD45HI CD11b+ Cells
*
VEH AAV2-SYN
FTY720 AAV2-SYN

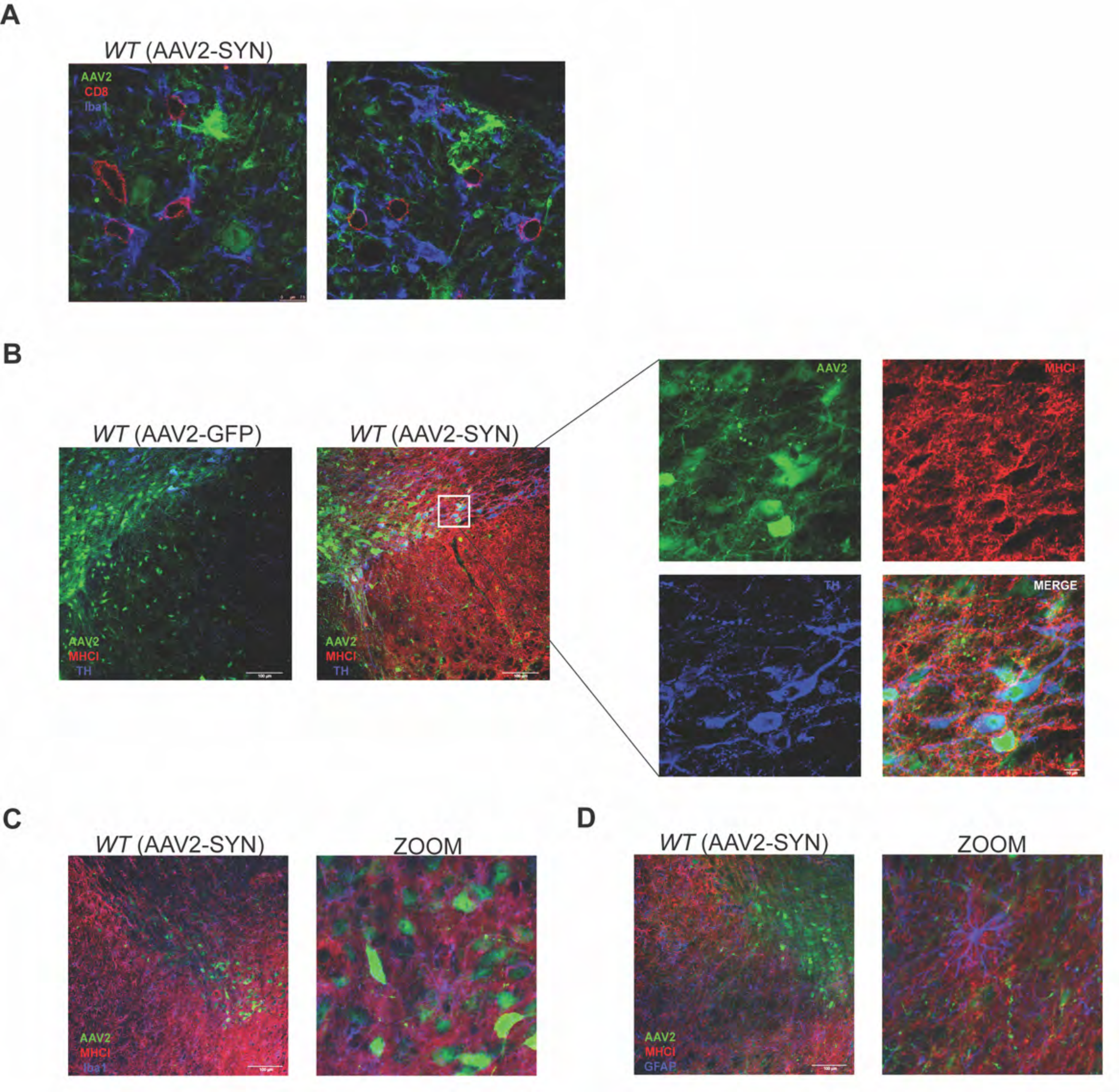
A
WT (AAV2-SYN)
AAV2
CD8
Iba1
B
WT (AAV2-GFP)
WT (AAV2-SYN)
AAV2
MHCI
TH
MERGE
C
WT (AAV2-SYN)
ZOOM
AAV2
MHCI
Iba1
D
WT (AAV2-SYN)
ZOOM
AAV2
MHCI
GFAP

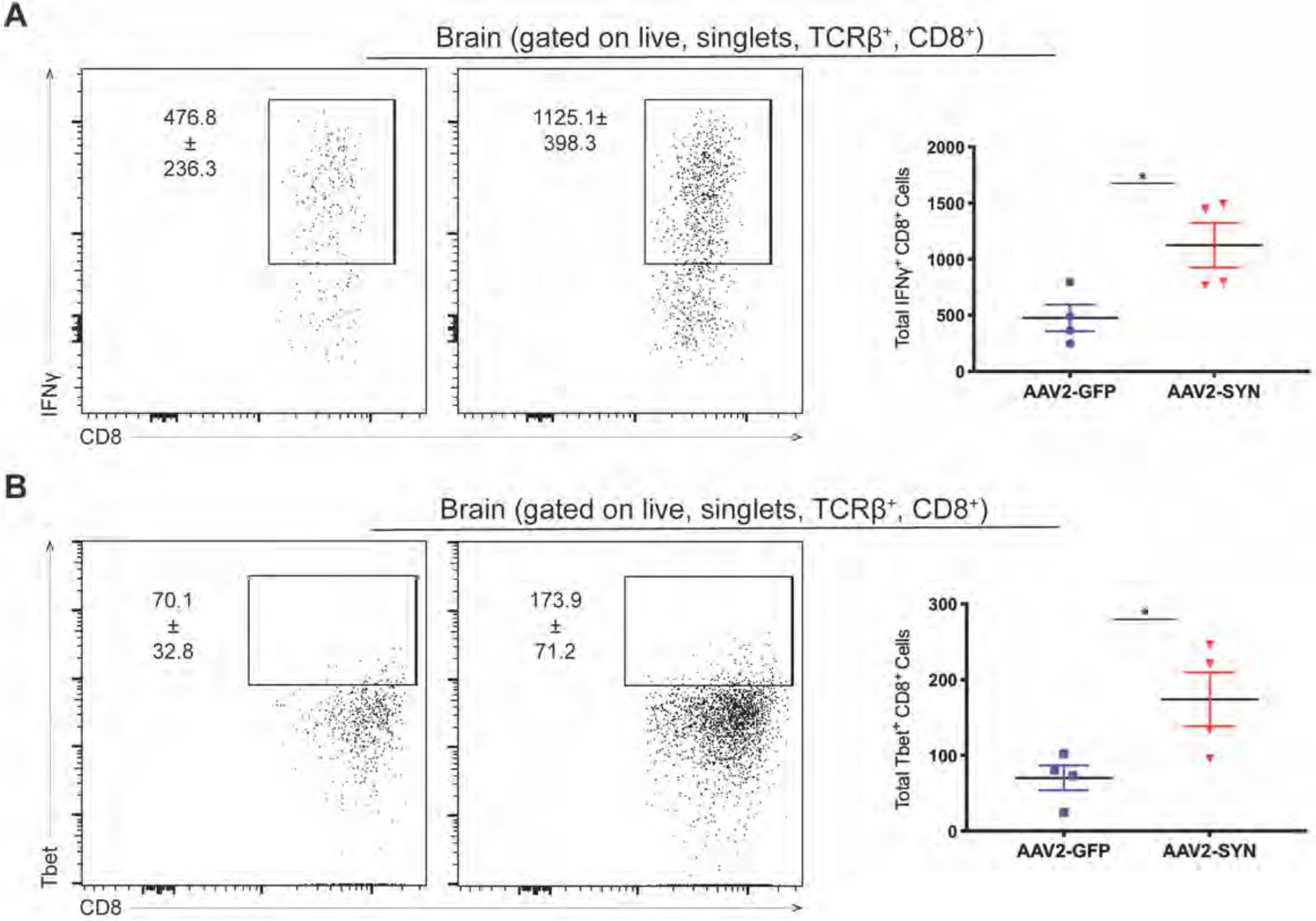
A
Brain (gated on live, singlets, TCRβ+, CD8+)
476.8
±
236.3
1125.1±
398.3
IFNγ
CD8
Total IFNγ+ CD8+ Cells
2000
1500
1000
500
0
*
AAV2-GFP
AAV2-SYN
B
Brain (gated on live, singlets, TCRβ+, CD8+)
70.1
±
32.8
173.9
±
71.2
Tbet
CD8
Total Tbet+ CD8+ Cells
300
200
100
0
*
AAV2-GFP
AAV2-SYN

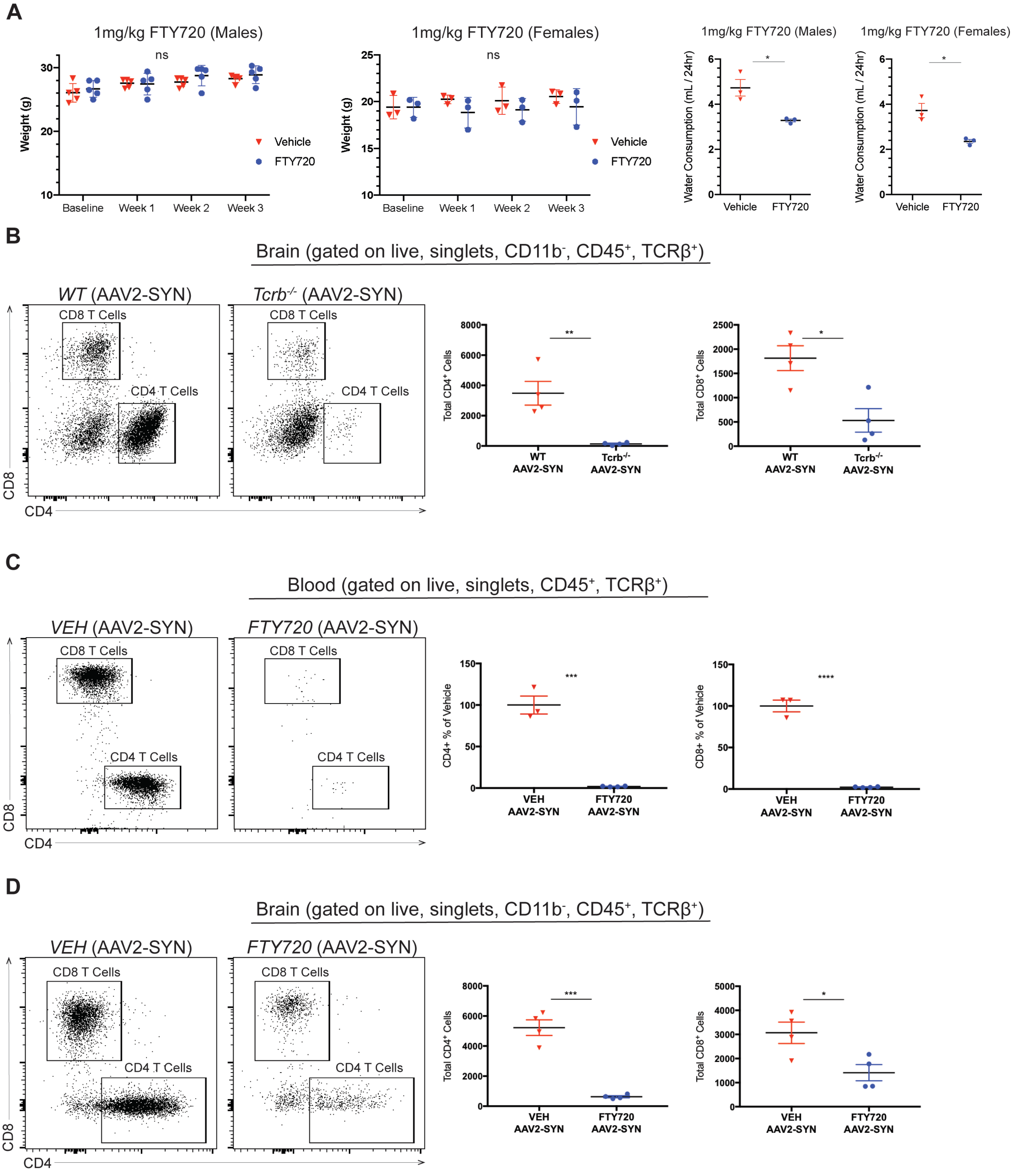
A
1mg/kg FTY720 (Males)
ns
Weight (g)
Vehicle
FTY720
Baseline
Week 1
Week 2
Week 3
1mg/kg FTY720 (Females)
1mg/kg FTY720 (Males)
1mg/kg FTY720 (Females)
Water Consumption (mL / 24hr)
B
Brain (gated on live, singlets, CD11b-, CD45+, TCRβ+)
WT (AAV2-SYN)
Tcrb-/- (AAV2-SYN)
CD8 T Cells
CD4 T Cells
CD8
CD4
Total CD4+ Cells
Total CD8+ Cells
WT AAV2-SYN
Tcrb-/- AAV2-SYN
C
Blood (gated on live, singlets, CD45+, TCRβ+)
VEH (AAV2-SYN)
FTY720 (AAV2-SYN)
CD4+ % of Vehicle
CD8+ % of Vehicle
VEH AAV2-SYN
FTY720 AAV2-SYN
D
Brain (gated on live, singlets, CD11b-, CD45+, TCRβ+)
VEH (AAV2-SYN)
FTY720 (AAV2-SYN)
Total CD4+ Cells
Total CD8+ Cells

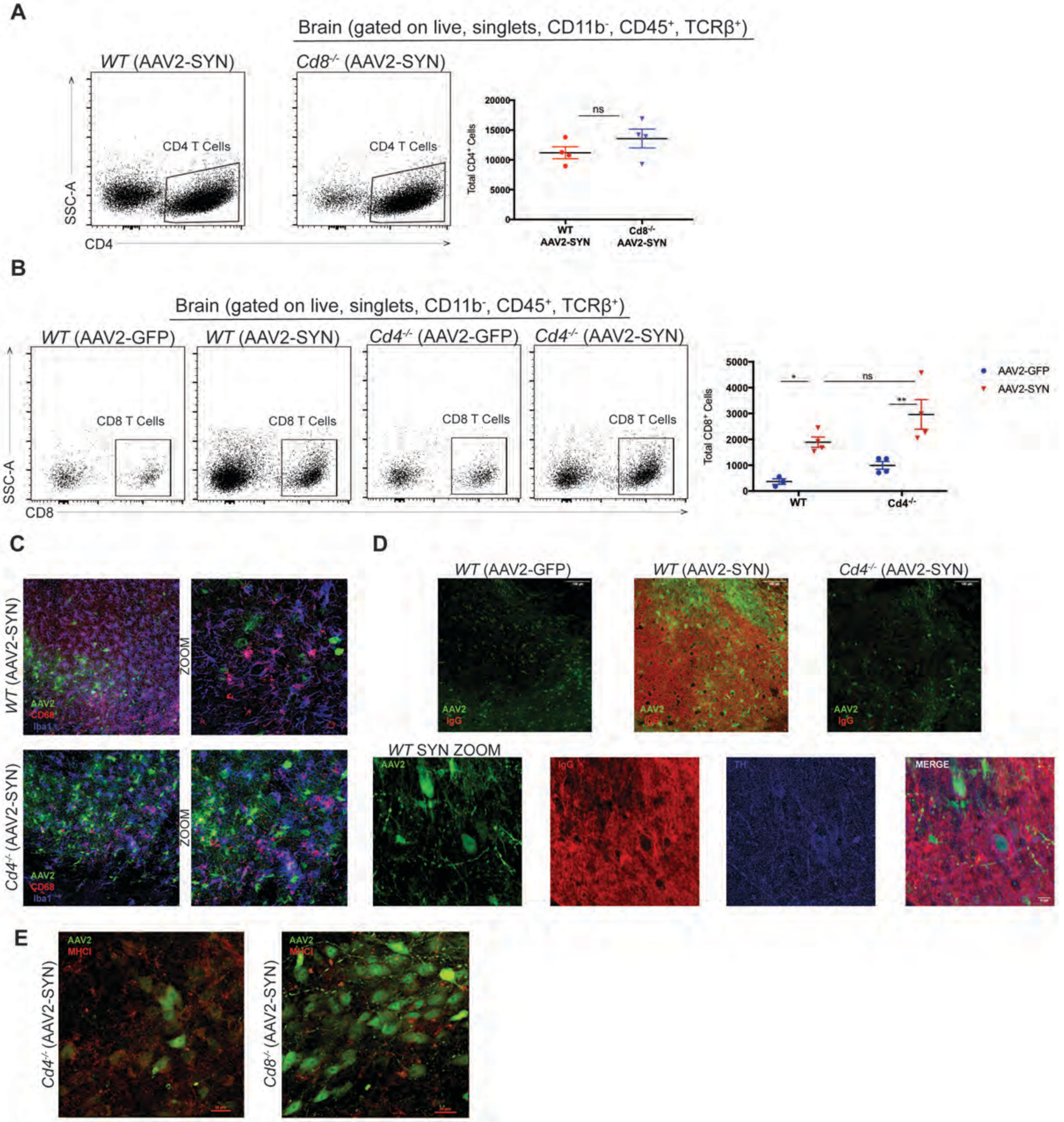

A
Brain (gated on live, singlets, CD11b-, CD45+, TCRβ+)
WT (AAV2-SYN)
Cd8-/- (AAV2-SYN)
CD4 T Cells
CD4 T Cells
SSC-A
CD4
Total CD4+ Cells
20000
15000
10000
5000
0
ns
WT
AAV2-SYN
Cd8-/-
AAV2-SYN
B
Brain (gated on live, singlets, CD11b-, CD45+, TCRβ+)
WT (AAV2-GFP)
WT (AAV2-SYN)
Cd4-/- (AAV2-GFP)
Cd4-/- (AAV2-SYN)
CD8 T Cells
CD8 T Cells
CD8 T Cells
CD8 T Cells
SSC-A
CD8
Total CD8+ Cells
5000
4000
3000
2000
1000
0
*
ns
**
WT
Cd4-/-
AAV2-GFP
AAV2-SYN
C
WT (AAV2-SYN)
Cd4-/- (AAV2-SYN)
ZOOM
ZOOM
AAV2
CD68
Iba1
AAV2
CD68
Iba1
D
WT (AAV2-GFP)
WT (AAV2-SYN)
Cd4-/- (AAV2-SYN)
AAV2
IgG
AAV2
IgG
AAV2
IgG
WT SYN ZOOM
AAV2
IgG
TH
MERGE
E
Cd4-/- (AAV2-SYN)
Cd8-/- (AAV2-SYN)
AAV2
MHCI
AAV2
MHCI
