# Supplementary material for: CD4 T cells mediate brain inflammation and neurodegeneration in a mouse model of Parkinson's disease
Source: Brain. 2021 Mar 11;144(7):2047–59. doi: 10.1093/brain/awab103 (PMC8370411; doi:10.1093/brain/awab103)
Supplement: awab103_Supplementary_Data [file awab103_supplementary_data.zip › brain-2020-01215-File009.pdf]

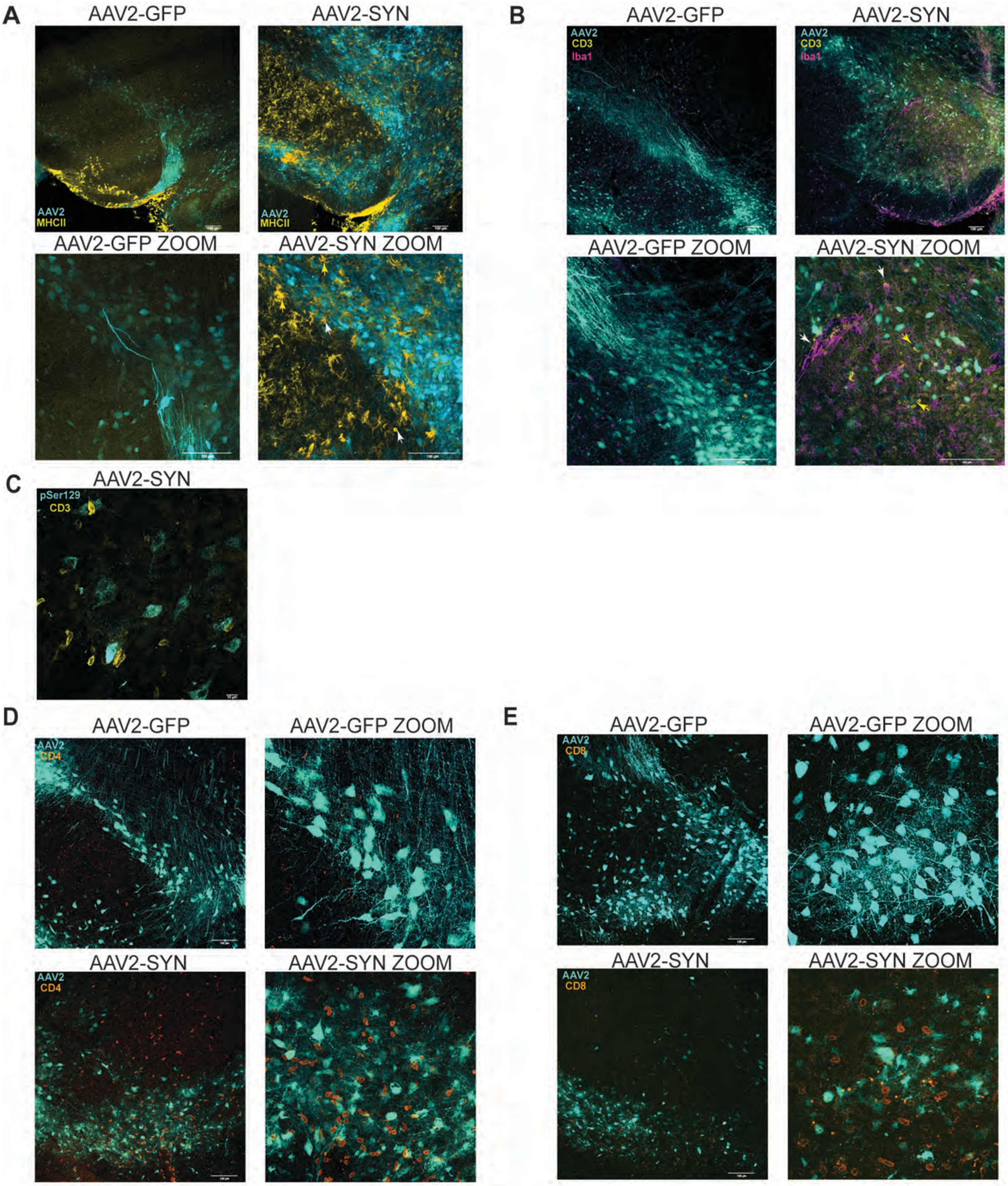
A
AAV2-GFP
AAV2-SYN
AAV2
MHCII
AAV2-GFP ZOOM
AAV2-SYN ZOOM
B
AAV2-GFP
AAV2-SYN
AAV2
CD3
Iba1
AAV2-GFP ZOOM
AAV2-SYN ZOOM
C
AAV2-SYN
pSer129
CD3
D
AAV2-GFP
AAV2-GFP ZOOM
AAV2
CD4
AAV2-SYN
AAV2-SYN ZOOM
E
AAV2-GFP
AAV2-GFP ZOOM
AAV2
CD8
AAV2-SYN
AAV2-SYN ZOOM

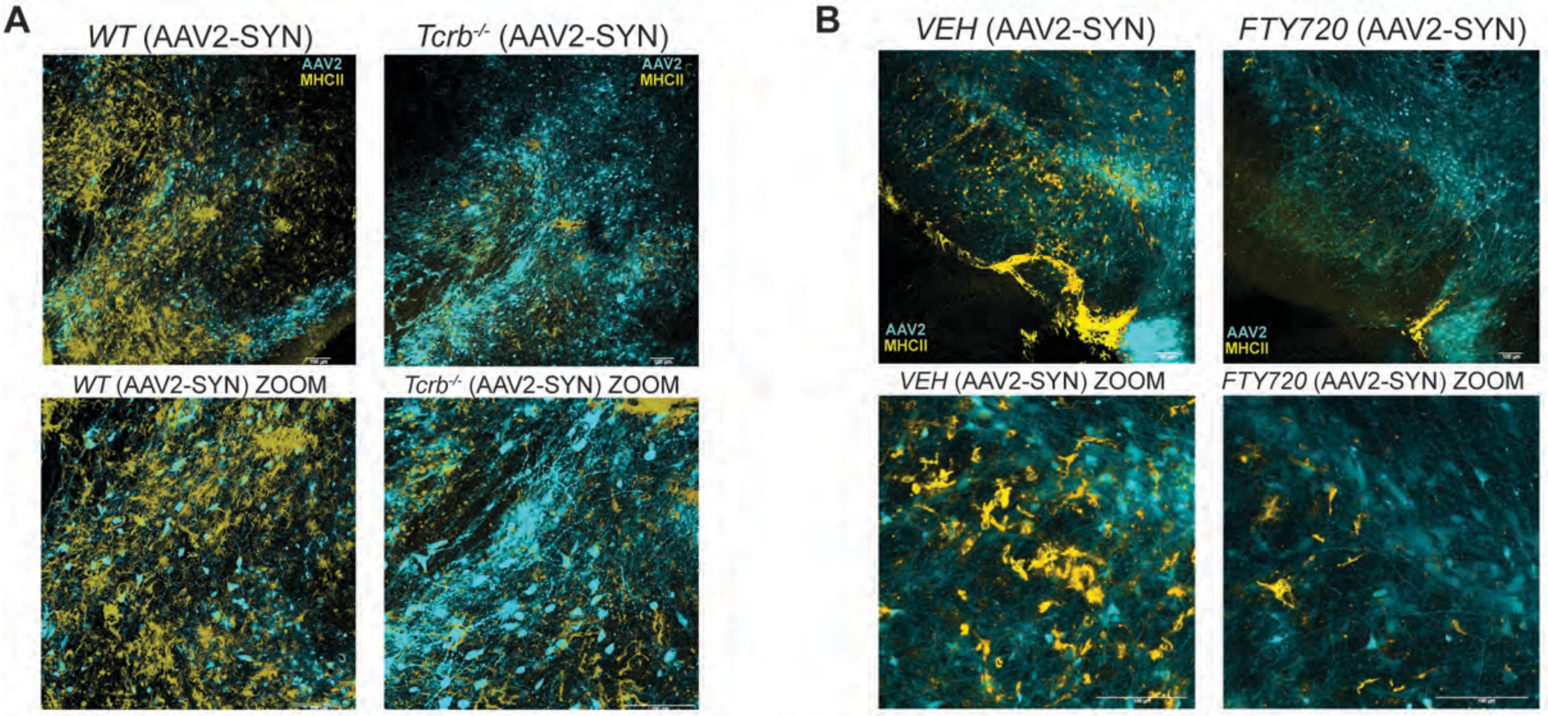
A
WT (AAV2-SYN)
AAV2
MHCII
Tcrb$^{-/-}$ (AAV2-SYN)
AAV2
MHCII
WT (AAV2-SYN) ZOOM
Tcrb$^{-/-}$ (AAV2-SYN) ZOOM
B
VEH (AAV2-SYN)
AAV2
MHCII
FTY720 (AAV2-SYN)
AAV2
MHCII
VEH (AAV2-SYN) ZOOM
FTY720 (AAV2-SYN) ZOOM

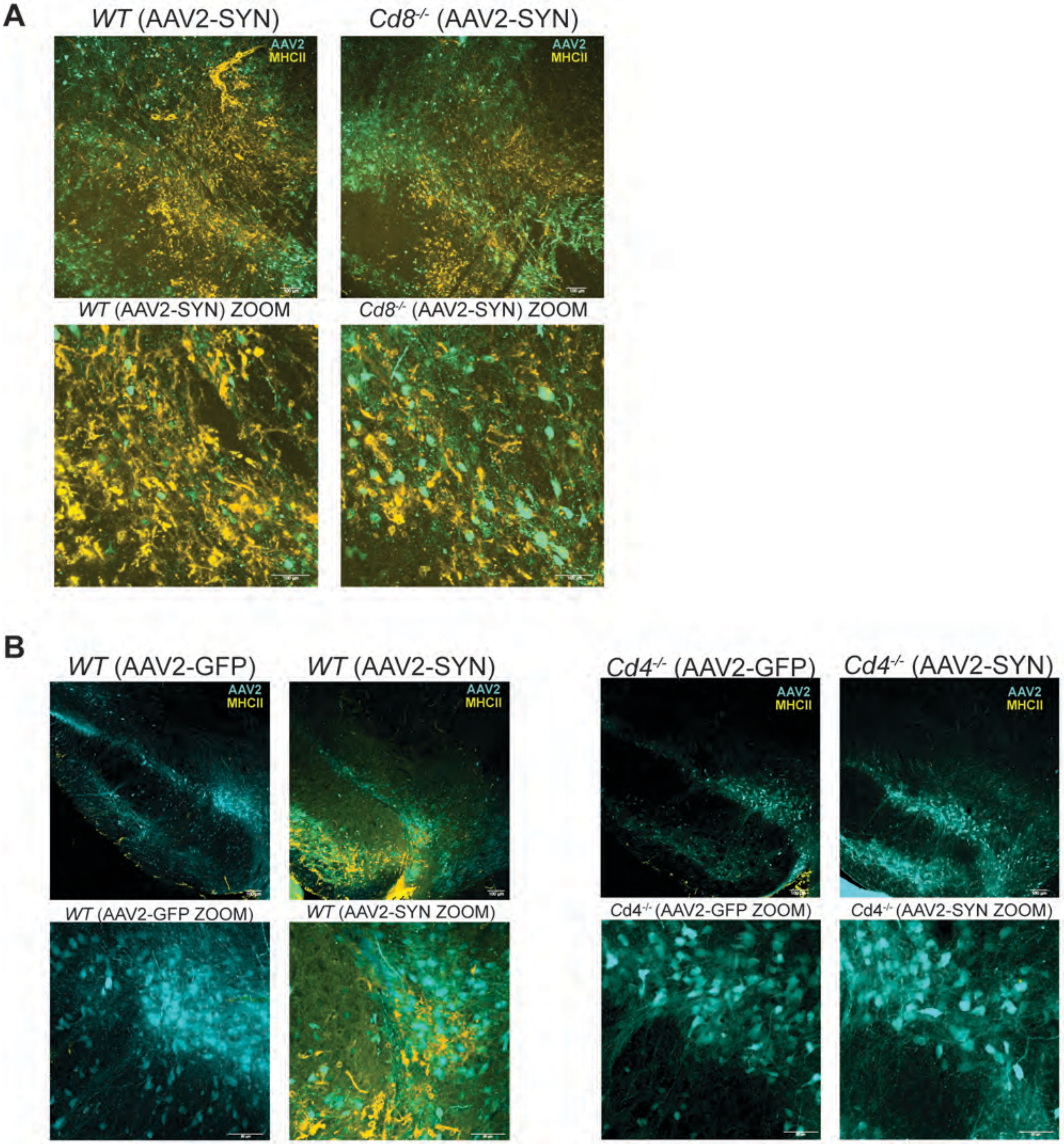
A
WT (AAV2-SYN)
Cd8-/- (AAV2-SYN)
AAV2
MHCII
WT (AAV2-SYN) ZOOM
Cd8-/- (AAV2-SYN) ZOOM
B
WT (AAV2-GFP)
WT (AAV2-SYN)
Cd4-/- (AAV2-GFP)
Cd4-/- (AAV2-SYN)
WT (AAV2-GFP ZOOM)
WT (AAV2-SYN ZOOM)
Cd4-/- (AAV2-GFP ZOOM)
Cd4-/- (AAV2-SYN ZOOM)
